# Pharmacologic and Non-Pharmacologic Interventions for HIV-Neuropathy Pain. A Systematic Review and a Meta-Analysis

**DOI:** 10.3390/medicina55120762

**Published:** 2019-11-28

**Authors:** Aikaterini Amaniti, Chrysanthi Sardeli, Varvara Fyntanidou, Panagiota Papakonstantinou, Ioannis Dalakakis, Antonios Mylonas, Konstantinos Sapalidis, Christoforos Kosmidis, Athanasios Katsaounis, Dimitrios Giannakidis, Charilaos Koulouris, Zoi Aidoni, Nikolaos Michalopoulos, Paul Zarogoulidis, Isaak Kesisoglou, Aris Ioannidis, Anastasios Vagionas, Konstantinos Romanidis, Panagoula Oikonomou, Vasilios Grosomanidis

**Affiliations:** 1 Anaesthesiology Department, “AHEPA” University Hospital, Aristotle University of Thessaloniki, Medical School, 54124 Thessaloniki, Greece; amanitik@gmail.com (A.A.); bfyntan@yahoo.com (V.F.); tsapap@gmail.com (P.P.); idalakakis@gmail.com (I.D.); vgrosoma@auth.gr (V.G.); 2 Department of Pharmacology & Clinical Pharmacology, School of Medicine, Faculty of Health Sciences, Aristotle University of Thessaloniki, 54124 Thessaloniki, Greece; sardeli@auth.gr (C.S.); antmilo.da@gmail.com (A.M.); 3 3rd Department of Surgery, “AHEPA” University Hospital, Aristotle University of Thessaloniki, Medical School, 54124 Thessaloniki, Greece; sapalidiskonstantinos@gmail.com (K.S.); dr.ckosmidis@gmail.com (C.K.); athanasios_katsaounis@hotmail.com (A.K.); giannakidis.d@gmail.com (D.G.); charilaoskoulouris@gmail.com (C.K.); nmichalopoulos@outlook.com (N.M.); issackesisoglou@outlook.com (I.K.);; 4 Intensive Care Unit, “AHEPA” University Hospital, Aristotle University of Thessaloniki, Medical School, 54124 Thessaloniki, Greece; zoiaidoni@yahoo.com; 5 Oncology Department, General Hospital of Kavala, 65500 Kavala, Greece; 6 Second Department of Surgery, University Hospital of Alexandroupolis, Medical School, Democritus University of Thrace, 25510 Alexandroupolis, Greece

**Keywords:** HIV, infectious disease, pain, neuropathy

## Abstract

*Background and Objectives:* Among HIV infection symptoms, sensory neuropathy (HIV-SN) remains a main cause of suffering, with incidence varying from 13–50%. So far, numerous pharmacological and non-pharmacological treatments have been tested, although few evidence-based analgesic options are available. We conducted an up-to-date systematic review and meta-analysis of the literature in order to evaluate the efficacy and safety of pharmacologic and non-pharmacologic treatments for pain control, in patients with HIV neuropathy. *Materials and Methods:* We searched MEDLINE, EMBASE, Scopus/Elsevier, The Cochrane Central Register of Controlled Trials (CENTRAL), USA Clinical Trials registry, and The International Web of Science up to April 2019. All randomized controlled trials evaluating efficacy and safety of non-pharmacologic and pharmacologic therapies were included. Efficacy was defined as pain reduction during the study period. Safety was estimated from adverse events. A meta-analysis was performed whenever possible. *Results:* 27 randomized controlled trials (RCTs) were included for analysis (7 evaluating non pharmacologic interventions, 20 pharmacologic therapies). Non-pharmacologic studies (n = 742) involved seven different therapeutic modalities. Only Acupuncture/Moxibustion showed pain reduction over placebo, Gracely Pain Scale Mean (SD): Acu/Moxa 0.85 (0.12), placebo 1.10 (0.09), *p* = 0.05. Pharmacologic studies, involving 2516 patients revealed efficacy for capsaicin 8% over placebo (mean difference −8.04 [95% CI: −14.92 −1.15], smoked cannabis (where pooling data for meta-analysis was not possible) and recombinant Nerve Growth Factor. *Conclusion:* Despite various modalities for pain control in HIV-SN, strongest evidence exists for capsaicin 8% and smoked cannabis, although of low methodological quality. Among non-pharmacologic modalities, only Acu/Moxa gave a marginal beneficial effect in one study, possibly limited by inherent methodological flaws.

## 1. Introduction

Among the broad spectrum of HIV infection symptoms, HIV sensory neuropathy (HIV-SN) remains one of the main causes of suffering, having subsequent impact on quality of life of these patients [1]. It has been estimated that up to one-third of HIV infected individuals suffer from HIV-SN [2], with incidence varying from 13% to up to 50% [3,4], primarily due to different diagnostic criteria [2,3]. HIV-SN is presented as distal symmetrical axonal, sensory polyneuropathy that primarily affects the feet, but it may also affect more proximal sites as well as the hands. While some of them are manifestations of the classic distal polyneuropathy, due to direct effect of HIV infection, others are caused by neuropathy due to antiretroviral therapy (ART), especially nucleoside analogue reverse-transcriptase inhibitors (NRTIs). Despite the fact that the two forms of neuropathy are caused by different pathophysiologic mechanisms [4], they share common and often indistinguishable clinical characteristics.

The main clinical characteristics of HIV associated polyneuropathy include pain, distal symmetrical burning sensation, paraesthesias, cramping in legs, muscle weakness, and increased fatigue. This kind of impairments may lead to psychological dysfunction, reduced quality of life and poor mobility. According to the literature, several therapies have been tested for palliative care that include analgesics, gabapentinoids, tricyclic antidepressants, membrane stabilizing factors, and non-pharmacological therapies as well. As distal symmetrical polyneuropathy has a negative impact on patients’ quality of life there is a great need to find effective pharmacological approaches to alleviate symptoms and manage pain.

Data regarding the exact prevalence of painful neuropathy among HIV patients are also quite variable in the literature. Evaluating pain syndrome in ambulatory AIDS patients, Hewitt et al. reported a 28% incidence of pain due to polyneuropathy among participants [5] Another study conducted by Tagliati et al. [6], found distal pain present in 38% among patients with distal polyneuropathy. In a more recent study, Adoukonou et al. revealed the presence of pain in 23.4% of patients [7], while other studies report painful symptoms in up to 75% of HIV-SN participants [8]. Pain is associated with depression and poor quality of life [1,9,10,11]. Depression seems to be associated with greater pain intensity [1,12].

Despite the high prevalence of painful neuropathy in patients living with HIV, pain is still undertreated [12,13]. Numerous pharmacological and non-pharmacological treatments have been used for alleviation of symptoms, although few evidence-based analgesic options for HIV-SN are available, based on clinical data [14]. A systematic review and meta-analysis of pharmacologic treatments conducted by Phillips et al. in 2010 showed evidence of efficacy only for capsaicin 8%, smoked cannabis and recombinant human nerve growth factor (rhNGF) [15].

During the time period from 2010 till know new randomized trials have been published examining the efficacy of various pharmacologic treatments [16,17] Furthermore studies on non-pharmacological treatments for HIV painful neuropathy are often seen in the literature. From this perspective, we conducted a systematic review and meta-analysis of the literature in order to evaluate the efficacy and safety of various pharmacologic and non-pharmacologic treatments in the alleviation of painful symptoms in patients with HIV neuropathy. The study included all published randomized studies comparing therapies with no therapies or with other therapies. This review examines the hypotheses that 1. The use of pharmacologic and non-pharmacologic interventions is effective in neuropathic pain alleviation, which characterize HIV-neuropathy. 2. Examined interventions are able to achieve meaningful improvement of painful symptoms, as defined by IMMPACT recommendations for the evaluation of reduction in pain [18] 3. The examined interventions are safe.

## 2. Methods

### 2.1. Protocol and Registration

**Figure 1:**
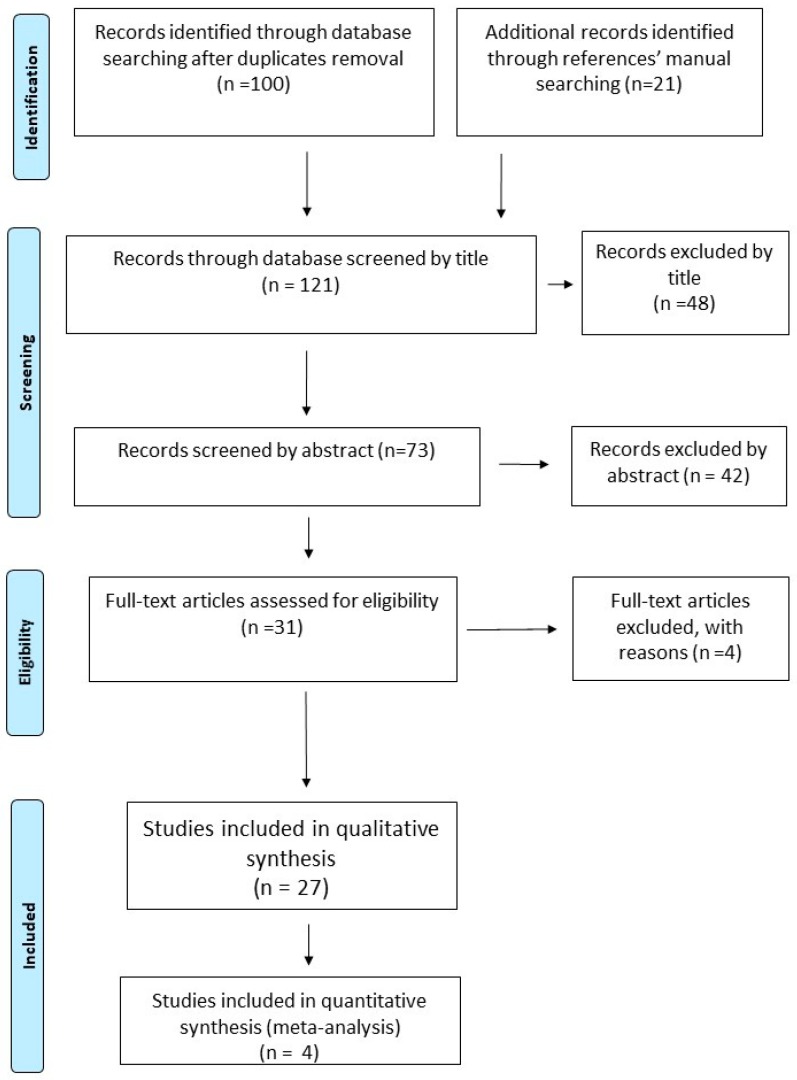
Flow diagram of the Study for the Systematic Review and Meta-analysis process [1].

Following the recommendations of the PRISMA statement for reporting Systematic Reviews and Meta-analyses, we conduct this systematic review and meta-analysis for studies examining the efficacy and safety of pharmacologic and non-pharmacologic treatments for painful HIV-sensory neuropathy [19]. The reviewing process was followed a specific predetermined protocol. The protocol can be accessed at PROSPERO, with registration number CRD42018084887 (https://www.crd.york.ac.uk/prospero/#searchadvanced). (Figure 1.)

### 2.2. Eligibility Criteria

Types of studies: Only randomized controlled trials (RCTs), studying pharmacologic and non-pharmacologic treatments for the management of painful HIV-neuropathy, were considered eligible for inclusion without any language or publication date restriction. Animal studies, reviews, letters, abstract-only trials, open-label trials, and trials that were not randomized were excluded from the study.

Types of participants: The study included patients >18 years old, infected with HIV virus and suffering from painful neuropathy. The latter was confirmed by the presence of symmetrical pain, burning, or dysesthesias in a stocking distribution with abnormal ankle reflexes or at least one abnormal sensory sign (elevated vibratory thresholds, reduced pinprick or temperature sensation, or cutaneous allodynia). Exclusion criteria were age < 18, pregnancy or breastfeeding, presence of renal or hepatic impairment, presence of diabetes or Vitamin B12 deficiency, treatment with known neurotoxic drugs and presence or other potential causes of neuropathy.

Types of interventions: RCTs examined pharmacologic or non-pharmacologic treatment for the management of painful HIV-neuropathy was eligible for inclusion. Any dosing scheme, formulation or route of administration was taken into consideration. Investigational intervention should has been compared to no treatment, placebo or sham treatment or other “active control” (alternative treatment).

Types of outcomes measures: The efficacy and safety of pharmacologic or non-pharmacologic treatments.

### 2.3. Primary Outcomes

Efficacy was confirmed by the reduction of pain measures in study population up to the end of study period. Furthermore, co-primary outcomes included the clinical efficacy, defined as percentage of patients with at least >30% pain reduction (IMMPACT recommendations for the evaluation of reduction of pain [18]. Safety was defined by the reports of side effects, which were directly attributable to the applied treatment.

### 2.4. Secondary Outcomes

Secondary efficacy outcomes included general improvement in clinical status, improvement in functional status, mood or sleep. Secondary safety outcomes included deaths or severe damage from the investigational intervention as well as number of discontinuation or withdrawal, possibly assigned to study intervention.

### 2.5. Systematic Search

The literature search was conducted in MEDLINE, EMBASE, Scopus/Elsevier, The Cochrane Central Register of Controlled Trials (CENTRAL), USA Clinical Trials registry (clinicaltrials.gov) and The International Web of Science databases up to 22 October 2018. The search used a combination of text words and MeSH, with no language restriction. Different searching strategy was followed for each database. The search strategy for MEDLINE is outlined in Appendix A. Additional search for possible recent literature was conducted in 10 April 2019. Also, the reference lists of the retrieved articles were manually searched for further relevant studies.

Based on the search strategy, all titles and abstracts retrieved were independently scanned by two authors (AA, ID). Each article retrieved was firstly assessed from the title or the abstract in order to evaluate whether fulfills eligibility criteria set. If eligibility could not be ascertained, based only on the title or the abstract, the full text of the study was retrieved and searched. The article was included for review if eligibility criteria were met, as judged by both authors. In case of disagreements between the two reviewers, a third author (PP) was responsible to resolve the disagreement and decide about the eligibility of the article. Interrater agreement was measured using Cohen’s kappa statistic [20].

A data collection sheet was created and included articles were assessed for:

1. Study design
2. Total study duration
3. Risk of bias (randomization if any, sequence generation, allocation sequence concealment, blinding of participants, personnel and outcome assessors, incomplete outcome data, selective outcome reporting and other concerns about bias).
4. Total number of participants
5. Diagnostic criteria for neuropathy, clinical (pain, burning, or dysesthesias in a stocking distribution, abnormal ankle reflexes or abnormal sensory signs) or electrophysiological
6. Age of participants.
7. Sex of participants.
8. Antiretroviral therapy.
9. Characteristics of interventions (treatment vs. placebo or treatment vs. other treatment).
10. Number of different intervention groups (Active treatment vs. placebo or other treatment).
11. Characteristics of treatment or intervention (dose, route of administration, duration).
12. Information about withdrawals.
13. Outcome measures (Pain, adverse events, discontinuation due to side effects).

### 2.6. Assessment of Risk of Bias

Internal validity of eligible studies was independently assessed by two review authors (AA and ID). Any disagreements between review authors were resolved by discussion. If consensus between authors could not be achieved, a third review author (PP) arbitrated to solve the disagreement.

Risk of bias assessment was conducted by using the ’Risk of bias tool’ of the Cochrane Handbook for Systematic Reviews of Interventions” [21]. Eligible trials were evaluated on the quality domains of random sequence generation, allocation concealment, blinding of participants and personnel, blinding of outcome assessors, incomplete outcome data addressed, selective reporting and any other source of bias as follows:

- Random sequence generation (selection bias): The method of allocation sequence generation was assessed as: low risk of bias (random number table, computer random generator) and unclear risk of bias (when the method is not clearly stated)
- Allocation concealment (selection bias): The procedure followed for avoidance of allocation foresight or changing. We assessed methods as low risk of bias (telephone or central randomization, consecutively numbered, sealed, opaque envelopes) or unclear risk of bias (when method is not clearly stated).
- Blinding of participants and personnel (performance bias): Methods for blinding participants and personnel were assessed and judged as low risk of bias (when study described in detail the method of blinding) and unclear risk of bias (when study stated it was blinded but did not provide adequate description of how this was achieved or didn’t report this domain).
- Blinding of outcome assessment (detection bias): we assessed the methods used to blind the allocated interventions by outcome assessors. We assessed methods as low risk of bias (study states blinding of outcome assessments ensured) or unclear risk of bias (when method is not clearly stated) and high risk (no blinding of outcome assessment).
- Incomplete outcome data (attrition bias): we assessed the methods used to deal with incomplete data as low risk of bias (when <10% of participants did not complete the study or if a “baseline observation carried forward” was followed), unclear risk of bias (when a “last observation carried forward” methodology was followed) or high risk of bias (when a per protocol analysis was followed).
- Selective reporting (reporting bias): We assessed methods as low risk of bias (all of the study’s pre-specified outcomes were reported and a protocol is available), unclear risk (insufficient information and no available protocol) or high risk (not all of the study’s pre-specified criteria were reported).

We considered a trial as having a low risk of bias only if all examined domains were judged as “low risk of bias”. Furthermore, we considered a study as having “high risk of bias” when one or more domain has been judged as “high risk of bias”.

### 2.7. Measures of Treatment Effect

We reported the primary outcomes of included studies as either a continuous variable (i.e., pain level reduction as estimated by validated numeric scales) or dichotomous variables (percent of patients with >30% pain reduction).

### 2.8. Assessment of Heterogeneity

Methodological and clinical heterogeneity was assessed, based on the characteristics of included studies (study design, parallel or cross-over, study population, inconsistency among interventions and treatment outcomes reporting). Statistical heterogeneity of included studies was assessed by visual inspection of the confidence intervals (CI) of forest plot results, estimations of *p* value and I^2^ statistic. *p* < 0.05 for χ^2^ test and I^2^ statistic >50% were considered as indicators for significant heterogeneity. The method of the random effects model was used for summarizing data in order to account for significant heterogeneity.

### 2.9. Sensitivity Analysis

In cases of significant statistical heterogeneity, we performed sensitivity analyses using both the fixed-effect and random-effects model. Furthermore, we intended to also perform sensitivity analysis by excluding studies with high risk of bias, whether these fulfil the criteria for quantitative analysis.

### 2.10. Assessment of Reporting Biases

If sufficient studies (at least 10) were identified, we assessed potential Publication biases using funnel plots asymmetry.

### 2.11. Data Synthesis

We based outcome data on intention-to-treat analysis results. We combined data from dichotomous and continuous outcomes and performed meta-analysis using Review Manager 5 when data from two or more RCTs were sufficient. For trials with multiple intervention groups, we combined groups to create single pair-wise comparisons as outlined in Chapter 16.5.4 of the Cochrane Handbook for Systematic Reviews of Interventions [21]. For dichotomous outcomes, we summed both the sample sizes and the numbers of people with events across groups, and for continuous outcomes, we combined means and standard deviations (SD) using the methods described in Section 7.7.3.8 of the Cochrane Handbook for Systematic Reviews of Interventions [21]. We used odds ratio (OR) to measure the treatment effect of dichotomous outcomes and the mean difference (MD) for continuous data using the inverse variance method. We used random-effect model, in order to account for heterogeneity among studies.

## 3. Results of the Search

Our initial search in MEDLINE, EMBASE, Scopus/Elsevier, The Cochrane Central Register of Controlled Trials (CENTRAL), USA Clinical Trials registry (clinicaltrials.gov) and The International Web of Science up to 22 October 2018 retrieved 100 potentially relevant articles after de-duplication. Furthermore, manual searching across references of these potentially relevant abstracts led to another 21 potentially relevant articles (Figure 1). Articles were firstly scanned by title by two independent searchers (AA and ID). From the 121 initially retrieved articles, 48 were excluded by title, leaving 73 possibly relevant articles to be scanned by abstract. Of these, 42 were excluded by abstract and the remaining 31 articles were assessed as full texts for eligibility. Among them, 27 original articles were considered as eligible, while 4 articles were excluded. Agreement between authors was quite substantial (Cohen’s k: 0.7973).

### 3.1. Excluded Studies

We excluded 4 studies for this review (Brown Simpson et al. [22], Silver et al. [23], Nazarbaghi et al. [24] and Penza et al. [25]) [22,23,24,25]. The study of Brown and Simpson was excluded because it was a review of two earlier published original articles. The studies of Silver et al. [23], Nazarbaghi et al. [24] and Penza et al. [25] were excluded because none of the patients received the study interventions had HIV neuropathy.

### 3.2. Included Studies

A total of 27 randomized controlled trials, examining pharmacologic and non-pharmacologic intervention for pain management in HIV neuropathy, were included for analysis. Among these studies, 6 were evaluated non-pharmacologic techniques for HIV neuropathy pain (Sandoval et al. [26], Paice et al. [27], Mkandla et al. [28], Maharaj et al. [29], Evans et al. [30], Anastasi et al. [31]), one examined a combination of pharmacologic and non-pharmacologic intervention (Shlay et al. [32]). 20 up to 27 studies examined pharmacologic administration in HIV neuropathy (Simpson et al. [33], Simpson et al. [34], Abrams et al. [35], Ellis et al. [36], Clifford et al. [37], Simpson et al. [38], Paice et al. [39], Simpson et al. [40], Simpson et al. [41], Dinat et al. [42], Kieburtz et al. [43], Kemper et al. [44], Hanh et al. [45], McArthur et al. [46], Youle et al. [47], Estanislao et al. [48], Simpson et al. [49], Evans et al. [50], Shiffito et al. [51], Harrison et al. [16]).

#### Non-Pharmacologic Studies

The seven included RCTs, evaluating non-pharmacologic techniques for HIV-neuropathy, involved a total of 742 participants. All non-pharmacologic studies followed a parallel design. A sham intervention was used as a control in all studies. One study (Shlay et al. [32]) followed 3 different enrolment modalities, a 2 × 2 factorial design (Acupuncture/Amitriptyline vs. Sham/Placebo), Acupuncture vs. Sham Acupuncture or Amitriptyline vs. placebo. One study (Maharaj et al. [29]), investigated two active interventions in comparison to sham intervention, while all the remained studies included one experimental arm. Characteristics of the included studies are shown in Table 1, Table 2 and Table 3.

### 3.3. Participants

Focusing on participants characteristics, 459/742 were men and 283/742 were women. Diagnosis of HIV neuropathy was clinical in all studies. Maharaj et al. [29] used the Brief Peripheral neuropathy screening tool for participants’ evaluation. No antiretroviral therapy or stable antiretroviral therapy as an entry criterion was considered in three studies (Maharaj 2018, Mkandla 2016, Anastasi 2013) [28,29,52], while two more studies provided antiretroviral therapy details of their participants in their results (Sandoval 2016, Evans 2003) [26,30]. The use of analgesics was liberal in all but Anastasi study, where a stable scheme for at least 8 weeks was considered for inclusion [52]. Data regarding baseline pain measurements couldn’t be pooled due to different measurement tools. A predefined baseline pain intensity level was used as a cut-off for enrolment only in Anastasi 2013 [52] and Evans 2003 [30] studies, with at least moderate level of pain as a prerequisite for inclusion.

### 3.4. Interventions

Among eligible studies, two examined Aerobic Exercise (AE) and Progressive Resisted Exercises (PRE) (Maharaj 2018, Mkandla 2016) [28,29], one studied Lower Extremity Splinting (LES) (Sandoval et al. [26]), one studied the combination of Acupuncture/Moxibustion (Acu/Moxa) (Anastasi et al. [31]), one studied Acupuncture plus Amitriptyline (Shlay et al. [32]), one study evaluated Cognitive Behaviour Therapy (CBT) versus Supportive Psychotherapy (SP) (Evans et al. [30]), while one trial studied Vibratory Stimulus (VS, Paice et al. [27]) Control interventions included HIV talks, video presentations, and counselling (Maharaj et al. [29]), usual care (Mkandla 2016, Evans 2003) [28,30] or sham intervention (Sandoval et al. [26], Anastasi et al. [31], Shlay et al. [32], Paice et al. [32]).

### 3.5. Outcomes

All studies, except for Mkandla et al. [28] (where Quality of life was the primary outcome), included pain measurement as a primary efficacy outcome. Different tools were used for pain measurement. Two studies used Numerical Rating Scale (NRS) 0–10 (Maharaj et al. [29]) and Neuropathy Pain Scale (NPS) 0–100 (Sandoval et al. [26]) for pain intensity estimation. Another two studies (Evans et al. [30], Paice et al. [27]) applied Brief Pain Inventory (BPI) while Anastasi et al. [52] and Shlay et al. [32] estimated primary outcome via Gracely Pain Scale (GPS). Data extraction and primary and secondary measures were made only for the longest follow-up period reported by the article. Three studies (Maharaj et al. [29], Mkdala et al. [28], Anastasi et al. [52]) followed patients for a period of 12 weeks and one study (Shlay et al. [32]) followed patients for 14 weeks. Another two studies restricted follow up period to six weeks (Sandoval et al. [26], Evans et al. [30]).

Regarding safety outcome, the studies of Maharaj et al. [29] and Mkdala et al. [28] didn’t report any side effect from the intervention, while the study of Anastasi 2013 [52] stated that side effects were mild. Sandoval reported 16/23 participants of the LES group suffering from discomfort with immobilization during the first 2 weeks of the trial, resolved by week 3, with no comfort-related issues reported by any of the participants in the liner group [27]. The study of Shlay et al. [32] reported side effects only in the arm received amitriptyline. Neither CBT nor SP lead to any side effect mentioned (Evans et al.) [30].

### 3.6. Risk of Bias of Included Non-Pharmacologic Studies

**Figure 2:**
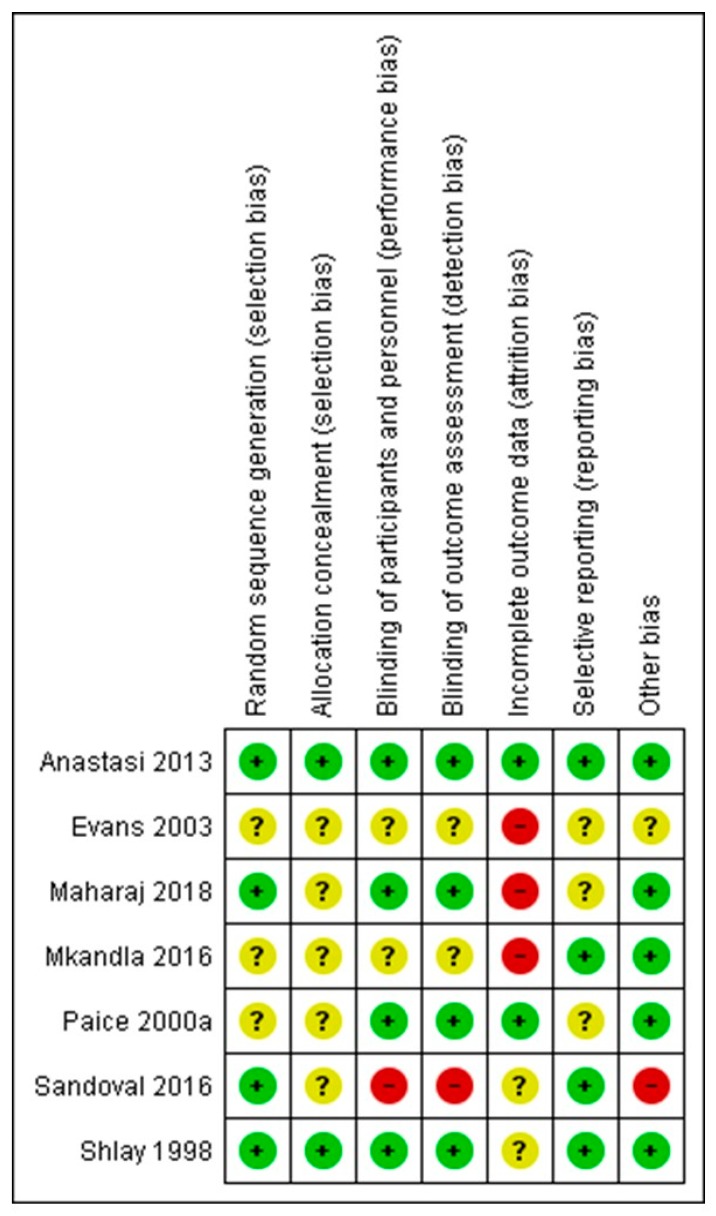
Review authors’ judgements about each risk of bias domain item for each included non-pharmacologic study (+ corresponds to low risk of bias, − corresponds to high risk of bias? corresponds to unclear risk of bias).

**Figure 3:**
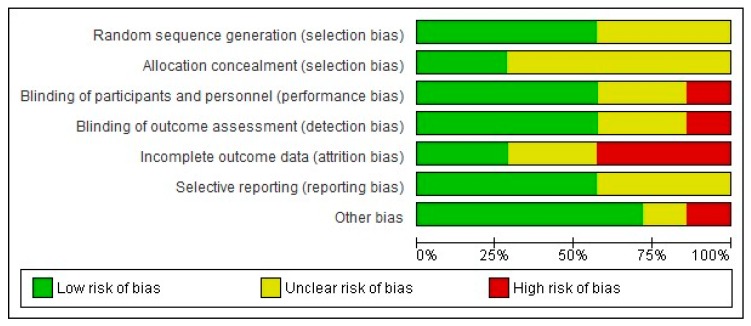
Graphical representation of the risk of bias in RCTs assessing the effects of non-pharmacological interventions in HIV neuropathy pain.

The authors’ judgments regarding all examined domains as well as graphical representation of overall results are shown in Figure 2 and Figure 3.

The risk of bias assessments identified that sequence generation and allocation concealment were often inadequately reported. Among the seven included studies, only four described the blinding protocol in detail (Maharaj et al. [29], Anastasi et al. [31], Shlay et al. [32], Paice et al. [27]). Regarding incomplete outcome data, the study of Maharaj et al. [29] followed per protocol analysis and not an intention to treat, despite the large proportion of dropouts (up to 10% dropouts without any data about differences in these patients, compared to patients that completed the protocol). Mkandla et al. [28], Evans et al. [30] and Sandoval et al. [26] trials are also characterised by a high dropout rate, raising the possibility of attrition bias. Finally, regarding possible selective reporting, participants, interventions and outcomes were possible to be compared with previously published protocols in cases of Anastasi et al. [31], Mkandla et al. [28] and Sandoval et al. [26] trials, without any violation from the published protocol, while for remained studies, data could not be retrieved. Consequently, only one study (Anastasi et al. [31]) was judged as of high quality (low risk of bias), while four studies (Maharaj et al. [29], Mkandla et al. [28], Sandoval et al. [26], Evans et al. [30]) were considered as carrying high risk of bias.

### 3.7. Aerobic Exercise (AE) and Progressive Resisted Exercises (PRE)

Two studies examined (AE) and (PRE). The study of Maharaj et al. [29] randomized patients to three groups to undergo AE, PRE or control intervention, while Mkandla et al. [28] examined PRE intervention compared to control intervention.

The trial undertaken by Maharaj et al. [29] examined the role of AE sessions or PRE sessions, provided 3 times a week for 12 weeks. The interventions were compared to a control intervention, including HIV talks, video presentations, and counselling. Pain intensity and distress were assessed with numeric pain rating scale from 0–10. Analysis regarding pain showed significant differences between groups at 12 weeks after intervention. Mkadla et al. [28] study followed a similar program of PRE exercises, with twice a week exercises for a study period of 12 weeks. In this study, control group just continued usual daily activities. While the primary outcome measure was quality of life, evaluated using The Shona version of the Euro Quality of Life-5 (Five) Dimensions (EQ-5D) state of health questionnaire, data about pain was possible to be extracted using pain/discomfort EQ-5D dimension. However, authors showed no statistically significant differences between PRE and control group in pain dimension except for the dimension of state of health (*p* = 0.04) and not pain. Pooled analysis of these two studies was not possible due to significant methodological heterogeneity, attributed to different outcomes and different pain estimation tools.

### 3.8. Acupuncture/Moxibustion (Acu/Moxa)

Anastasi 2013 randomized 50 HIV patients to receive either true Acupuncture/Moxibustion or sham Acupuncture/Placebo Moxibustion for 12 twice weekly session and a total follow up period of 15 weeks [32] The assessment of lower-limb pain was made using the GPS, as a primary outcome and the Subjective Peripheral Neuropathy Screen (SPNS). Acu/Moxa group showed significantly reduced pain rates at the end of 15 weeks, compared to baseline (Baseline Means (SE): 1.21 (0.04), follow up week 15: 0.85 (0.12), *p* < 0.05). Sham/placebo group also showed significant reduction in pain at week 15, compared to placebo (Mean (SE): Baseline 1.30 (0.04), follow up week 15: 1.10 (0.09), *p* < 0.05). Between groups comparisons showed significantly reduced pain scores for Acu/Moxa group compared to placebo at week 15 (*p* < 0.01).

Shlay 1998 evaluated the effect of Acupuncture (Standard Acupuncture regimen, SAR) plus Amitriptyline vs. sham/placebo, as well as the effect of either intervention (SAR or Amitriptyline) vs. their matching sham/placebo treatment [32] SAR/Sham arm, 114 randomized patients underwent twice weekly sessions of either SAR or sham treatment for a total of 14 weeks. GPS changes from baseline didn’t demonstrate any significant differences between groups (mean difference CI: −0.08 (CI: −0.21 to 0.06), *p* = 0.26.

### 3.9. Lower Extremity Splinting (LES)

One study (Sandoval et al. [26]), enrolled 46 patients, examining the effect of Walkabout splints (LES), compared to sham splints (Liner only) in HIV neuropathy. Patients wore the splints or liners during night sleep for 6 weeks. At the end of follow up period, pain and sleep were evaluated through Neuropathic Pain Scale 0–100 The Pittsburgh Sleep Quality Index (PSQI) 0–21 questionnaires. Analysis of results revealed that while both interventions improve pain and sleep over time, neither was superior in the domains of pain reduction and sleep improvement (*p >* 0.05). [26].

### 3.10. Cognitive Behaviour Therapy (CBT) vs. Supportive Psychotherapy (SP)

The study (Evans et al. [30]) recruited 61 patients to undergo week sessions of CBT or SP for 6 weeks. The examined parameters included pain (BPI average pain intensity), depression, generalized anxiety, phobic anxiety, somatization, hostility, and interpersonal sensitivity (Brief Symptom Inventory), interference with functioning, Distress (Beck Depression Inventory, Hamilton Depression Rating Scale) and function (Karnofsky Performance Scale). Regarding primary outcome measures, both the CBT group and the SP groups showed significant reductions in measures of pain intensity. However, these differences were not statistically significant.

### 3.11. Vibratory Stimulus (VS)

The study conducted by Paice et al. [27] was the only one that examined the immediate analgesic effect of applied vibratory stimulus. After a session of 45 min, patients didn’t show immediate improvement in pain intensity, compared to sham intervention.

## 4. Pharmacologic Interventions

Systematic searching identified twenty RCT, examining different pharmacologic regimen for HIV DSN [27,34,35,36,37,38,39,40,41,42,43,44,45,46,47,48,50,51,53], plus one study (Shlay et al. [32]), evaluating Acupuncture plus Amitriptyline effect. The follow up period varied from 5 days to 18 weeks. Among 20 included pharmacologic studies, five followed a cross-over design (Estanislao et al. [48], Ellis et al., Dinat et al. [42], Kemper et al. [44] and Harrison et al. [16]) and the remaining a parallel design. Despite the fact that review attempted to include only ITT patients’ data, it wasn’t always possible for these data to be retrieved. This was particularly the case for cross-over studies with multiple comparisons and dropouts between arms. Consequently, cross-over studies underwent only qualitative and not quantitative analysis, in order to avoid methodological pitfalls when pooling with parallel design studies. Characteristics of the included studies are shown in Table 4, Table 5, Table 6, Table 7, Table 8, Table 9, Table 10, Table 11 and Table 12.

### 4.1. Participants

The 20 eligible pharmacologic studies enrolled 2516 patients). The vast majority of patients were male (The study of Estanislao et al. [48] didn’t report data about subjects’ sex). The majority of studies included subjects with either no current use of ART or stable dose of ART for at least 4 weeks before enrolment. The studies performed by Ellis et al. [36] and Shiffito et al. [51] didn’t include ART therapy status in the inclusion criteria. Regarding missing data management only one study followed a baseline observation carried forward practice (Simpson et al. [54]), while last observation carried forward method was used in 9 studies (Clifford et al. [37], Simpson et al. [33], Simpson et al. [38], Evans et al. [50], Youle et al. [47], Simpson et al. [40], McArthur et al. [46], Kieburtz et al. [43], Simpson et al. [49]) and a per protocol analysis in 5 (Dinat et al. [42], Harrison et al. [16], Abrams et al. [35], Simpson et al. [41], Kemper et al. [44]).

### 4.2. Interventions

Fifteen different drugs were evaluated for efficacy and safety, including Amitriptyline (Dinat et al. [42], Shlay et al. [32], Kieburtz et al. [43]), Pregabalin (Simpson et al. [54], Simpson et al. [53]), Duloxetine and Methadone (Harrison et al. [16]), Capsaicin (Clifford et al. [37], Simpson et al. [38], Paice et al. [27,39]), Smoked cannabis (Ellis et al. [36], Abrams et al. [35]), Prosaptide (Evans et al. [50]), L-Carnitine (Youle et al. [47]), Memantine (Shiffito et al. [51]), Lidocaine gel (Estanislao et al. [48]) Gabapentin (Hanh et al. [45]), Lamotrigine (Simpson et al. [40], Simpson et al. [41]), NGF (McArthur et al. [46]), Mexiletine (Kieburtz et al. [43], Kemper et al. [44]) and Peptide T (Simpson et al. [49]). All studies, except for Harrison et al. [16] and Kieburtz et al. [43], used a placebo group for comparison. Cross-over studies included a washout period between study arms.

### 4.3. Outcomes

All studies included pain as a primary efficacy outcome. Thirteen studies (Hahn et al. [45], Youle et al. [47], Simpson et al. [53], Simpson et al. [34], Abrams et al. [35], Ellis et al. [36], Dinat et al. [42], Kemper et al., Shiffito et al. [51], Harrison et al. [16], Simpson et al. [38], Clifford et al. [37], and Paice et al. [35,39]) evaluated pain using some type of a Likert 0–10 scale. Eight studies (McArthur et al. [46], Estanislao et al. [48], Evans et al. [50], Simpson et al. [49], Kieburtz et al. [43], Simpson et al. [40], Simpson et al. [41], Shlay et al. [32]) measured efficacy regarding pain change with the Gracely Pain Scale (GPS) tool. One study (Ellis et al. [36]) also applied Descriptor Differential Scale (DDS) measurement in the primary outcome measures, apart from Likert 0-10 scale.

The outcome of percentage of patients with at least >30% pain reduction was reported in 6 trials (Abrams et al. [35], Simpson et al. [53], Simpson et al. [34], Evans et al. [50], Simpson et al. [38], Clifford et al. [37]).

Secondary measures include Patient/Clinical Global Impression of Change (P/CGIC, Youle et al. [47], Simpson et al., Simpson et al. [34], Simpson et al. [38], Clifford et al. [37]), sleep (Hahn et al. [45], Simpson et al. [53], Simpson et al. [34]) and mood (Abrams et al. [35], Ellis et al. [36], Kieburtz et al. [43], Paice et al. [39]).

## 5. Risk of Bias in Included Studies

**Figure 4:**
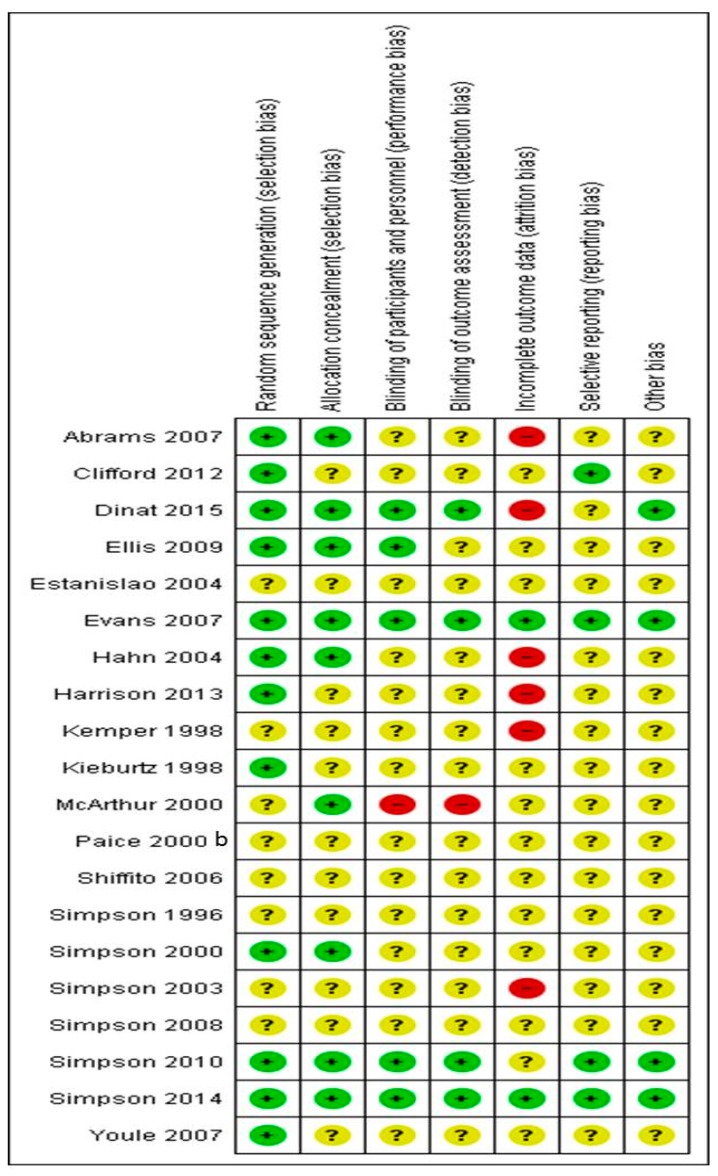
Review authors’ judgements about each risk of bias domain item for each included pharmacologic study (+ corresponds to low risk of bias, − corresponds to high risk of bias, ? corresponds to unclear risk of bias).

**Figure 5:**
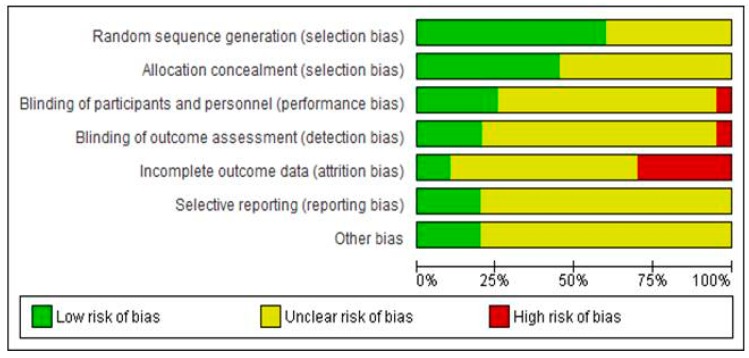
Graphical representation of the risk of bias in RCTs assessing the effects of pharmacological interventions in HIV neuropathy pain.

We assessed the risk of bias in included studies (Figure 4: review authors’ judgements about each risk of bias item for each included study and Figure 5, (risk of bias graph: risk of bias items presented as percentages across all included studies).

## 6. Allocation (Selection Bias)

Twelve out of 20 studies reported a reliable technique for randomization (Abrams et al. [35], Clifford et al. [37], Dinat et al. [42], Ellis et al. [36], Evans et al. [50], Hahn et al. [45], Harrison et al. [16], Kieburtz et al., Simpson et al. [40], Simpson et al. [33,44], Simpson et al. [34], Youle et al. [47]) and judged as low risk of bias for randomization. The remaining didn’t describe the method of randomization and are judged as unclear risk of bias. No study described a non-acceptable technique of randomization.

## 7. Allocation Concealment (Selection Bias)

Only nine studies described in detail the method of allocation concealment of participants and are judged as low risk of bias for the specific domain (Abrams et al. [35], Dinat et al. [42], Ellis et al. [36], Evans et al. [50], Hahn et al. [45], McArthur et al. [46], Simpson et al. [38], Simpson et al. [53], and Simpson et al. [34]). The remained eleven studies gave no information about allocation concealment (Unclear risk of bias).

## 8. Blinding of Participants and Personnel (Performance Bias and Detection Bias)

Five studies (Dinat et al. [42], Ellis et al. [36], Evans et al. [50], Simpson et al. [53], and Simpson et al. [34,43]) described in detail the method of blinding and assessed as low risk of bias. In one study (McArthur et al. [46]) blinding was unmasked due to active treatment side effects (High risk of bias). The remained 14 studies gave no information about allocation concealment (Unclear risk of bias).

## 9. Blinding of Outcome Assessment (Detection Bias)

Four studies (Dinat et al. [42], Evans et al. [50], Simpson et al. [33], and Simpson et al. [34]) reported the method of blinding of outcome assessors (Low risk of bias), while the remained 16 studies didn’t report this domain in detail (unclear risk of bias).

## 10. Incomplete Outcome Data (Attrition Bias)

Only two studies (Evans et al. [50], Simpson et al. [54]) followed an Intent to treat analysis with a “baseline observation carried forward” approach for missing data and judged as low risk of bias. On the other hand, Abrams et al. [35], Dinat et al. [36,42], Hahn et al. [45], Harrison et al. [16], Simpson et al. [41] trials managed missing data by following a “per protocol analysis” (high risk of data). The remained 13 studies followed an “intent to treat” analysis, with a “last observation carried forward” methodology for missing data (unclear risk of bias).

## 11. Selective Reporting (Reporting Bias)

A predefined protocol was available and available to access in four trials (Clifford et al. [37], Evans et al. [50], Simpson et al. [53], and Simpson et al. [34]), where predefined criteria and outcomes were reported in the final article (low risk of bias). In all other cases. Selective reporting biases were judged as unclear, due to no published trial protocol. However, all outcomes described in the methods section were fully reported in results.

Based on the above assessment, only two studies were considered as having low risk of bias (Evans et al. [50], Simpson et al. [34]), while six studies were considered as having high risk of bias (Abrams et al. [35], Dinat et al. [42], Hahn et al. [45], Harrison et al. [16], Kemper et al. [44], Simpson et al. [55]).

## 12. Pregabalin

Two studies (Simpson et al. [53], Simpson et al. [34]) [34,35] examined the role of Pregabalin in HIV-SN pain, including 679 patients, randomized to Pregabalin or matching placebo administration. The two studies followed similar methodology, with Pregabalin doses titrated to 600 mg in 2 and 4 weeks respectively and maintained for 12 weeks thereafter. Primary efficacy outcomes were the change in pain, measured with NRS (0–10), at the end of follow-up period and the responder rates of patients with >30% and 50% pain reduction. Regarding secondary efficacy outcomes, both studies examined Patient and Clinician Global Impression of Change (PGIC/CGIC), pain change using Brief Pain inventory short form (BPI sf) and Neuropathic Pain Symptom Inventory (NPSI), pain-related sleep interference and overall sleep disturbance with the NRS-Sleep scale and the Medical Outcomes Survey (MOS) sleep scale. Mood and anxiety symptoms were evaluated using the Hospital Anxiety and Depression Scale (HADS). Simpson et al. [33] also evaluated pain using Gracely Pain Scale (GPS), while Simpson et al. [34] study examined the impact of symptoms on functional activity, work productivity, and quality of life using the Work Productivity and Activity Impairment Specific Health Problem Questionnaire (WPAI-SHP), and the Short-Form 36 Health Survey (SF-36).

### 12.1. Primary Efficacy Outcomes

**Figure 6:**
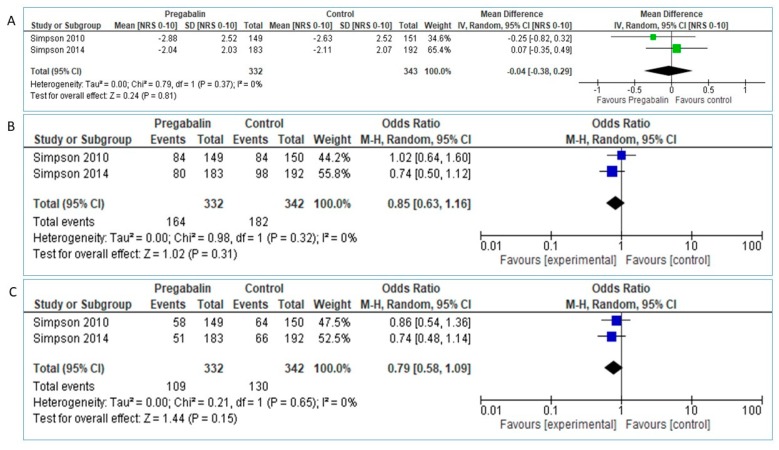
(**A**). Pregabalin vs. placebo, NRS [0–10] reduction (**B**). Pregabalin vs. placebo, proportion of patients with >30% NRS reduction. (**C**). Pregabalin vs. placebo, proportion of patients with >50% NRS reduction.

The analysis of pooled data in 332 Pregabalin and 343 placebo patients didn’t reveal superiority of Pregabalin over placebo in NRS pain reduction between baseline and study endpoint: MD = −0.04 [95% CI:−0.38, 0.29], test for overall effect: Z = 0.24 (*p* = 0.81, Figure 6A).

The Chi^2^ and I^2^ value reveal statistical homogeneity between studies. Heterogeneity: Tau^2^ = 0.00; Chi^2^ = 0.79, df = 1 (*p* = 0.37); I^2^ = 0%. However, we proceeded to sensitivity analysis of the results, by further applying fixed-effect model. Analysis of the results showed the same effect (MD = −0.04 [95% CI: −0.38, 0.29], Heterogeneity: Chi^2^ = 0.79, df = 1 (*p* = 0.37); I^2^ = 0%, Test for overall effect: Z = 0.24, *p* = 0.81).

Regarding primary outcome of proportion of patients with >30% pain reduction, pooled data from 332 Pregabalin and 342 placebo patients showed slight but not significant superiority of Pregabalin over placebo, OR: 0.85 [95% CI:0.63, 1.16], Heterogeneity: Tau^2^ = 0.00; Chi^2^ = 0.98, df = 1 (*p* = 0.32); I^2^ = 0%, Test for overall effect: Z = 1.02 (*p* = 0.31, Figure 6B).

Applying fixed-model the results were similar: Chi^2^ = 0.98, df = 1 (*p* = 0.32); I^2^ = 0%, Test for overall effect: Z = 1.02 (*p* = 0.31).

Similarly, pooled data regarding proportion of patients with >50% pain reduction didn’t show statistically significant superiority of Pregabalin over placebo: OR 0.79 [95% CI: 0.58, 1.09], Test for overall effect: Z = 1.44 (*p* = 0.15), Figure 6C.

Using fixed-effect model also lead to similar results: Chi^2^ = 0.21, df = 1 (*p* = 0.65); I^2^ = 0%, Test for overall effect: Z = 1.44 (*p* = 0.31).

### 12.2. Secondary Efficacy Outcomes

No significant differences were reported by either study, regarding the outcomes of PGIC/CGIC, BPI sf, NPSI, NRS-Sleep scale, MOS sleep scale, HADS, GPS, WPAI-SHP or SF-36.

### 12.3. Safety

Both studies reported Pregabalin treatment as being generally well tolerated. The most commonly reported AEs were somnolence, dizziness, euphoric mood, headache, and peripheral edema, while no treatment-related serious AEs occurred (Table 4).

### 12.4. Smoked Cannabis

Two studies, involving 89 patients, compared cannabis cigarettes with inactive cigarettes, containing 0% delta-9-THC (Abrams et al. [35], Ellis et al. [36]). Abrams et al. followed a double blind parallel design, while Ellis et al. [36] conducted a placebo-controlled, crossover trial. The concentration of active substance delta-9-THC was fixed in Abrams et al. [35] (delta-9-THC 3.56%), while Ellis et al. [36] titrated smoked delta-9-THC concentrations to effect (delta-9-THC 1–8%). Patients were followed up to the end of treatment arms (5 days) where primary and secondary endpoints were obtained (Table 5).

### 12.5. Primary Efficacy Outcomes

Both studies examined change in pain magnitude between baseline measurements and end of treatment period (5 days) measurements, as well as proportion of patients with >30% pain improvement. Methodological heterogeneity (parallel vs. cross-over trial, different tools for pain measurement, i.e., VAS vs. DDS), precluded proper pooling of data. However, both studies reported significantly greater pain reduction with cannabis, compared to placebo, as well as greater proportion of patients with >30% pain reduction in cannabis arm, compared to placebo arm (Table 5). However, both studies are characterized by poor methodological quality (high and unclear risk of bias for Abrams et al. and Ellis et al. [36] trials respectively).

### 12.6. Secondary Efficacy Outcomes

Additional variable estimated by both trials included the change in total mood disturbance (Profile of Mood States, POMS). Ellis et al. [36] also evaluated Disability, mood, and quality of life using Sickness Impact Profile (SIP) tool, the Brief Symptom Inventory (BSI) and a subjective Highness/Sedation Scale. Abrams measured immediate changes in chronic neuropathic pain VAS rating after 1^st^ and last cigarette and areas of secondary hyperalgesia produced by the heat/capsaicin sensitization model to brush and von Frey hair stimuli. All secondary parameters examined didn’t reveal significant differences between treatment groups except for immediate effect of first and last cigarette, where a reduction of chronic pain ratings, compared to placebo (*p* < 0.001) were recorded. Active cannabis also marginally reduced the area to both brush and von Frey hair stimuli compared to placebo (median −34% vs. −11%; *p* = 0.05 and −52% vs. +3%; *p* = 0.05).

### 12.7. Safety Outcomes

Confusion, dizziness, nausea, concentration difficulties, fatigue, sleepiness or sedation, increased duration of sleep, reduced salivation, and thirst were significantly more frequent in cannabis group, compared to placebo. However, Abrams et al. [35] study didn’t report any AE related dropouts, possibly attributed to treatment. On the other hand, 2 patients withdrew from Ellis study, as presented with psychosis and cough while being on cannabis arm (Table 5).

### 12.8. Capsaicin

Three studies (Clifford et al. [37], Simpson et al. [38], Paice et al. [39]), including 827 patients, evaluated capsaicin versus placebo for HIV-SN pain. Among them, two (Simpson et al. [38], Clifford et al. [37]) compared capsaicin 8% dermal patch capsaicin 640 μg/cm^2^, 8% *w*/*w*), as an active treatment, vs. placebo patch, for a follow up period of 12 weeks. The other study Paice et al. [39]) compared topical capsaicin cream (0.075%), as an active drug to placebo cream, for a follow up period up to 4 weeks.

## 13. Primary Efficacy Outcome

**Figure 7:**
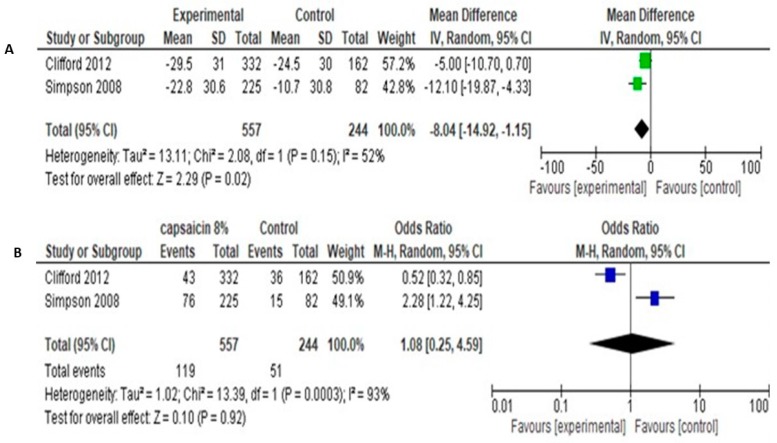
(**A**). Capsaicin 8% over placebo, NRS 0–100 reduction, random effect. (**B**). Capsaicin 8% over placebo, proportion of patients with >30% NRS reduction.

The studies of Simpson et al. [38] and Clifford et al. [37] involved a total of 557 patients in experimental group and 244 patients in control group. The both used the primary outcome of NRS change (0–100) in pain intensity from baseline to end of follow up (12 weeks). Methodological and clinical heterogeneity was considered quite low, in order to proceed to quantitative analysis. Pooled analysis of data showed that the Mean Difference in NRS pain change between baseline-12 weeks was −8.04 [95% CI: −14.92 −1.15] (Figure 7A).

Secondary outcome was the percentage of patients achieving >30% pain reduction, as a cut-off for clinical significance. Pooled data showed odds ratio of 1.08 [95% CI: 0.25, 4.59] and no significant differences between groups (Test for overall effect: Z = 0.10, *p* = 0.92, Heterogeneity: Tau^2^ = 1.02; Chi^2^ = 13.39, df = 1, *p* = 0.0003, I^2^ = 93%). Pooled results are shown in Figure 7B.

Due to significant statistical heterogeneity between studies, we performed sensitivity analyses using both the fixed-effect and random-effects models. Fixed models reveal quite similar results with random models, regarding both NPRS change (Mean difference −7.48 [95% CI: −12.08, −2.89], Test for overall effect: Z = 3.19 (*p* = 0.001) and proportion of patients with >30% pain reduction (Odds ratio 0.97 [95% CI: 0.67, 1.41], Test for overall effect: Z = 0.15 (*p* = 0.88).

**Figure 8:**
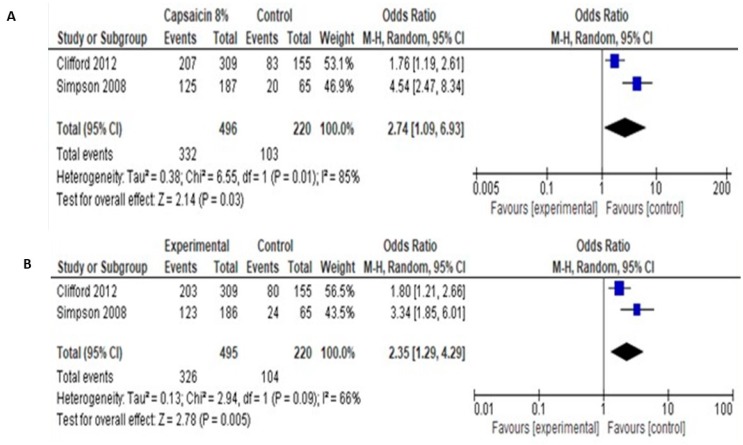
(**A**). Capsaicin 8% over placebo, PGIC. (**B**). Capsaicin 8% over placebo, CGIC.

Regarding PGIC and CGIC, pooled data showed significantly more patients showing very much, much or slight improvement, compared to control (odds ratio 2.74 [95% CI: 1.09, 6.93] for PGIC and 2.35 [95% CI: 1.29, 4.29] for CGIC (Figure 8).

Safety: Clifford et al. [37] recorded a 93% of Capsaicin group patients and 83% of control group patients having at least one AE. Respective percentages were 72% and 55% in Simpson et al. [38] study. Most of them were related to application site conditions. No deaths were considered as related to treatment (Table 6).

One trial of capsaicin cream was included in the review (Paice et al.). 26 participants, diagnosed with HIV-SN by their physician, randomized to apply capsaicin cream 0.075% or inactive cream, 4 times daily for 4 weeks. By using BPI as a primary measure of pain, capsaicin cream 0.075% group showed slight inferiority, over inactive cream (Mean (SD): Capsaicin 5.50 (2.68), Control 3.10 (2.12)) only at the end of week 1. Other secondary outcomes measured (QLI, POMS, SIP, touch–pressure sensation), didn’t reveal any differences between groups in any time point.

### 13.1. Lamotrigine

Two trials conducted by Simpson et al. (Simpson et al. [40] and Simpson et al. [41], Table 7) examined the potential efficacy and safety of Lamotrigine (LTG) over placebo [41,42]. The first trial (Simpson et al. [40]) included 42 patients (20 LTG group, 22 placebo group) who were randomly assigned to receive LTG or matching placebo. The dosing regimen started at 25 mg/day, titrated up to 150 mg twice per day after 7 weeks and maintained thereafter up to week 14. The second study (Simpson et al. [41]) randomized 227 patients (150 LTG, 77 placebo) to receive LTG or placebo. Here, the dosing regimen included a starting dose of 25 mg LTG or matching placebo every other day or 25 mg daily, based on the co-administration of drugs, known to induce the metabolism of LTG. After a 7-week LTG (and placebo) dose escalation phase, patients entered a 4-week maintenance phase, administered the target maintenance dose of LTG of 400 mg/day (for those not receiving enzyme-inducing drugs) and 600 mg/day (for those receiving enzyme-inducing drugs).

Both studies shared primary efficacy measure, defined as the mean change in average pain as measured by the Gracely Pain Scale (GPS) from baseline to the end of the maintenance phase. Furthermore, both studies followed a subgroup analysis, based on the concomitant use of ARTs. Regarding the first study (Simpson et al. [40]), per protocol analysis of primary outcome results showed a superiority of LTG, over placebo in pain reduction from baseline to study endpoint (Mean Difference (SE): Placebo −0.18 (0.09), LTG −0.55 (0.14), *p* = 0.03, which however, was not apparent when an intent to treat analysis was conducted. Subgroup analysis showed a significant difference only on patients not exposed to neurotoxin. However, the latter study conducted by the same team (Simpson et al. [41]), enrolling a greater number of subjects, didn’t demonstrated superiority of LTG over placebo, in any of the ART-expose subgroups. Despite the similar methodology and outcomes, pooling of data was not possible, since authors in Simpson et al. [41] study didn’t report any measure of variation in their data but only group mean values.

### 13.2. Amitryptiline and Mexiletine

One cross-over study examined the effect of Amitriptyline over placebo [42]. Authors randomized 124 HIV patients (62 ART naive patients and 62 ART users) to four subgroups to receive amitriptyline first, then placebo or placebo first, then amitriptyline. Patients titrated up to 150 mg amitriptyline (based on tolerance and effect) and matching placebo. Each treatment period consisted of 6 weeks with a washout period of 3 weeks between treatment periods. Analysis of results regarding efficacy, showed no significant differences in the absolute change in pain score over six weeks of treatment with placebo or amitriptyline in the ARV-user group, the ARV-naïve, or all participants combined (Table 8). However, the study revealed a significant period effect. When data from the ARV-user and ARV-naïve groups were combined and taking into consideration only first period, there was a significant decrease in pain intensity in both treatment groups over the six-week period, without any between groups difference. Safety parameters revealed that amitriptyline was safe and caused no resinous side effects. The most frequent side effects in both arms were drowsiness, dry mouth and chest pain, which were common to the use of Amitriptyline and placebo, with dry mouth complain more mentioned in Amitriptyline arm (*p* < 0.01).

The study of Shlay et al. [32] involved a pharmacologic arm, including 71 patients receiving Amitriptyline 25–75 mg daily and 65 patients receiving placebo for a period of 14 weeks. Primary endpoint of pain reduction was measured via GPS. Analysis showed that Amitriptyline was not superior to placebo regarding pain reduction (mean difference 0.00, CI: −0.18 −0.19, *p* = 0.99).

Kieburtz et al. [43] randomized 145 patients to receive a combination to active amitriptyline/placebo mexiletine, active mexiletine/placebo amitriptyline, or active control amitriptyline/placebo mexiletine. Intervention lasted 8 weeks (4 titration phase, 4 stable dose) and maximum possible doses of 100 mg amitryptiline and 300 mg of mexiletine. Therapeutic effect was measured using the Gracely Pain Scale (GPS) 0–1.77. Authors concluded that were no significant changes in the measures of pain intensity among the treatment groups (*p* = 0.38). Safety analysis showed that while no drug was related to laboratory or ECG changes, amitriptyline was associated with sedation requiring dosage modification in 10 subjects, and mexiletine was associated with nausea and vomiting, requiring dosage modification, also in 10 patients (Table 8).

The study of Kemper et al. [44] compared the analgesic effect of mexiletine over placebo in a randomized cross-over study, involving 22 HIV patients. Mexiletine or matching placebo was titrated to up to 600 mg/day (or 300 mg/day if the higher dose was not tolerated). The duration of each treatment arm was 6 weeks, with an interval washout period of 1 week, whereas the efficacy outcome was the change in VAS score. Analysis of each group showed that either administration of mexiletine followed by placebo or administration of placebo followed by mexiletine, lead to significant differences in VAS scores. Furthermore, analysis of mexiletine administration over placebo administration, irrespective of the order administered, also didn’t show significant differences between arms (*p* = 0.76). 39% of patients in mexiletine arm experienced side effects leading to dose reduction or discontinuation (rush, 1 patient, gastrointestinal side-effects, 2 patients).

### 13.3. Gabapentin (GBP)

A RCT conducted by Hahn et al. [45] evaluated the efficacy and safety of GBP in managing pain in HIV-SN. 26 eligible patients randomized to receive GBP or matching placebo, titrated to 1200 mg/day over 2 weeks, or up to 2400 mg/day if effect was considered insufficient, during the subsequent 2 weeks. Patients were evaluated regarding efficacy with 0-10 VAS scale of SF-McGill, and sleep interference score, measured by VAS (0 = excellent sleep, 10 cm = no sleep). Although both arms showed reduction in pain between baseline measurements and 4 week measurements, the difference between groups was not significant for either pain or sleep. However, GBP use was associated with more AE (dizziness, gait ataxia, and nausea, Table 9).

### 13.4. Recombinant Human NGF (rhNGF)

Only one trial (McArthur et al. [46]), enrolling 270 patients, examined the effects of recombinant human NGF (rhNGF). Investigators, evaluated the effects of 2 different dosing regimen 0.1 mg/kg rhNGF s.c. 2 times/week or 0.3 mg/kg rhNGF, s.c. 2 times/week versus placebo s.c. 2 times/week, for a period of 18 weeks. Study’s primary efficacy endpoint was the change in self-reported, average daily pain intensity) measured with Gracely Pain Scale (GPS) from baseline to week 18. A significant difference among treatment groups was noted for changes in average and maximum pain intensity from baseline to week 18, favouring rhNGF (Table 9). Authors mentioned site pain was the most frequent adverse event, resulting in violation of blinding in 39% of subjects, while severe transient myalgic pain occurred in eight patients, usually from accidental overdosing.

### 13.5. Acetyl L-Carnitine

One RCT examined the potential symptomatic treatment of antiretroviral toxic neuropathy by acetyl L-carnitine (ALCAR, Youle et al. [47]). 90 HIV patients, exposed to nonnucleoside reverse transcriptase inhibitors (NNRTIs), nucleoside reverse transcriptase inhibitors (NRTIs) and protease inhibitors (PIs), were randomized to receive acetyl L-carnitine (ALCAR) 1000 mg/day i.m., twice daily for 14 days (double blind period), follow by an open phase period. Considering efficacy, patients were estimated by VAS, Total Symptom Score (TSS), Clinical Global Impression of Change score (CGI-C) and McGill. In the ITT population ALCAR group showed greater reduction in pain, compared to placebo, measured by VAS, but this difference wasn’t statistical significant. Similarly, differences recorded through TSS, CGIC and McGill were comparable between groups. Reports of AE included 20.9% of ALCAR patients with at least one AE vs. 29.8% in the control arm (Table 10).

### 13.6. Lidocaine

Estanislao et al. [48] conducted a randomized, double blinded, cross over study to examine the efficacy of lidocaine gel 5% in HIV-DSN. 64 patients were ramdomized to apply 5% lidocaine gel or vehicle gel, once daily for 2 weeks (Phase A), followed by a 2 weeks washout period and then entered a second 2 weeks’ intervention period (Phase B, vehicle gel or lidocaine 5% gel application). The primary outcome was the difference in average pain scores, measured with Gracely pain scale (GPS) between the 2 groups during the second week of each treatment period, while secondary outcomes included differential effect of the first treatment, difference in global pain relief, and pain response by neurotoxin exposure. Regarding primary efficacy outcome, analysis of changes in pain scores between baseline and end of 2 weeks treatment phase didn’t show significant differences for either lidocaine or placebo (*p* = 314 for Phase A and *p* = 0.714 for Phase B respectively).

### 13.7. Peptide T

Simspon et al. [49] evaluated the efficacy of intranasally administered peptide T (6 mg per day), compared to placebo, in a randomized, double blind, parallel group study. The study enrolled 81 patients, who receive the study or placebo intervention for a period of 12 weeks. The primary measure of clinical efficacy was reduction in pain severity, measured by GPS at study end (week 12), while the secondary outcome measures assessed were neurologic examination, nerve conduction studies, global evaluation, electrophysiologic measurements, cognitive function and immunological function. Primary efficacy analysis (per protocol analysis) showed that there were no significant differences In the reduction in mean pain score for either treatment group at week 12 compared to baseline (*p* = 0.3). Furthermore, none of the secondary outcomes measured showed any differences between the two groups examined. There was no significant difference in adverse events between the two groups. The only event as possibly drug-related was a placebo recorded group patient mild epistaxis in one patient in the placebo group (Table 11).

### 13.8. Prosaptide

One study (Evans et al. [50]) evaluated the efficacy and safety of Prosaptide (PRO) for the treatment of painful HIV-associated sensory neuropathy. 229 patients were randomized to receive subcutaneously (S.C.) either PRO 2 mg/day, PRO 4 mg/day, PRO 8 mg/day, PRO 16 mg/day or placebo (PBO) for 6 weeks. The primary efficacy endpoint was the 6-week change from baseline in the weekly average of evaluable daily random prompts measuring pain using the GPS. Secondary endpoints included ‘‘treatment success’’, defined as >0.35 units of pain improvement from baseline on the Gracely scale, and change in HIV viral load. Safety endpoints included treatment emergent serious adverse events (SAEs), AEs, and toxicities. Analysis of data showed that after 6 weeks of treatment, changes from baseline in GPS ware comparable for all treatment PRO arms, compared to PBO. Moreover, treatment success rates were also comparable (19%, 28%, 22%, 28% and 22% for 2, 4, 8, 16 PRO arms and PBO arm respectively). Regarding possible adverse effects, 4 were observed in patients receiving PRO and were considered as possibly unrelated to treatment (cellulitis, shigella enteritis, altered mental status, and pancreatitis). Likewise, 1 side effect, possibly unrelated to treatment was recorded in PBO cohort (Table 11).

### 13.9. Memantine

A randomized, placebo controlled, parallel group, double blind study, conducted by Shiffito et al., examined the potential efficacy role of memantine in HIV-SN related pain. [51] 45 patients were randomized to receive memantine or matched placebo. Dose regimen started at 10 mg/day, titrated up to 40 mg, based on tolerance, and remained stable up to 16 weeks (primary study endpoint). Efficacy analyses examined the change in pain and paresthesia indices on a 01-10 scale, from baseline to week 16, between memantine and placebo arms. Neither pain nor paresthesia pain change measurements showed significant differences between groups. Authors mentioned no difference in adverse experiences between the two groups during the trial, without any other detail (Table 12).

### 13.10. Duloxetine, Methadone

A trial conducted by Harrison et al. [16] randomized 15 patients to a cross over design with 4 treatment sequence, containing the following arms: duloxetine/placebo, placebo/methadone, duloxetine/methadone, placebo/placebo (Table 12). Each treatment arm was applied for 4 weeks, with 1 week intervals between arms. The primary outcome measure was mean pain intensity at the end of each arm, measured on a 0 = 10 Likert scale where 0 = “No pain” to 10 = “Pain as bad as you can imagine”. Secondary outcome measure included night-time pain intensity. Authors concluded that no differences in the fourth week mean pain scores were detected between any of the active treatments and placebo, or between combination duloxetine-methadone and duloxetine or methadone monotherapy. Significant pair-wise differences were not either detected in night-time MPI scores between treatments. Safety analysis revealed no deaths, life-threatening adverse events (AEs), or severe laboratory abnormalities during the study. Overall, 4 patients reported 5 AEs while on duloxetine, 6 participants reported 17 grade AEs while on methadone, 5 patients reported 17 AEs while on combination therapy and 5 participants reported 6 AEs while on placebo. Severe adverse events on duloxetine included nausea (n = 1), vomiting (n = 1), renal dysfunction (n = 1). Severe adverse events on placebo included pain (n = 1) and fatigue (n = 1).

## 14. Discussion

The analysis included 27 studies, involving six different non-pharmacologic interventions and 15 different drugs. Hence, most pharmacologic and non-pharmacologic interventions have been examined by either one or very few studies, including small number of participants. Moreover, with few exceptions, whenever more than one study examined a specific intervention, pooling of data was impossible, primarily due to profound methodological and clinical heterogeneity. This is clearly obvious, regarding the different tools and questionnaires used for pain assessment. Lack of conformity in trials design, negatively contributes to revealing possible evidence for significant efficacy.

Among the non-pharmacologic studies, only two, examining Aerobic exercise (AE), Progressive Resisted Exercises (PRE) (Maharaj et al. [29]) and Acupuncture/Moxibustion (Acu/Moxa) (Anastasi et al. [52]) reported a small but statistically significant effect, regarding the primary efficacy outcome of pain reduction. However, the reported beneficial effect should be interpreted with caution. Despite the possible beneficial effect of Aerobic exercise (AE) and Progressive Resisted Exercises (PRE) over sham intervention (Maharaj et al. [29]), the specific study is judged as having “high risk of bias”, due to large number of dropouts and a “per protocol analysis’ of missing data. On the other hand, the study of Anastasi 2013 was considered of higher methodologic quality, regarding risk of bias. However, even this study suffers from other methodologic pitfalls as it has a small sample size. Furthermore, the use of GPS for pain evaluation, without a concomitant VAS or NRP scale, makes extremely difficult the interpretation of results, considering the clinical significance of this pain reduction. Consequently, among non-pharmacologic studies, only (Acu/Moxa) intervention seemed to be promising, necessitating the conduct of well-designed RCTs.

Similarly, among the twenty pharmacologic studies, only six revealed a statistical significant effect attributed to smoked cannabis, lamotrigine, capsaicin (Abrams et al. [35], Ellis et al. [36], Clifford et al. [37], Simpson et al. [38], Simpson et al. [40], McArthur et al. [46]). The beneficial effect of capsaicin, and NGF (Clifford et al. [37], Simpson et al. [38], Abrams et al. [35], Ellis et al. [36], McArthur et al. [46]) has been stated in the previous meta-analysis of Philips et al. [15]. Moreover, the beneficial effect of smoked cannabis also reported by Phillips meta-analysis, who, after obtaining data from the authors, estimated the pooled RR for >30% pain reduction at 2.38 (95% CI: 1.38 to 4.10). Based on these data, emerging evidence for smoked cannabis efficacious effect in pain reduction also complies with IMMPACT recommendations [18] regarding pain measurement and clinical significance. However, apart from problems orienting from combining data from parallel and cross-over trials, both studies lack long term follow up. The reported beneficial effect is demonstrated for up to five days. HIV sensory neuropathy is a chronic condition. Under this point of view a follow up period of such a short magnitude, poses considerable questions about the overall efficacy and safety of this intervention to alleviate painful symptoms [56]. Despite these problems in study design, the emerging interesting in medicinal cannabis, under the view of legalization of its use, makes urgent the conduct of well-designed RCT, with special attention to its long term effects. Finally, one small study (Simpson et al. [40]) demonstrated a positive effect for lamotrigine, over placebo. Nevertheless, this positive effect was detected when patients completed the study period were analysed. Similar results were not apparent when an intent to treat analysis, with a last observation carried forward method was used. Furthermore, this study has a considerable small sample size, with no data about power analysis. Taking all this data under consideration, the efficacious effect of lamotrigine should be considered rather questionable. The latter is supported by the absence of a positive effect in the subsequent well-designed large study of Simpson et al. [41]

During the period 2010–2018, new RCTs were published, examining the potential efficacious effects of various pharmacologic interventions. Among them, four (Dinat et al. [42], Clifford et al. [37], Harrison et al. [16], Simpson et al. [34]) met the inclusion criteria to be analysed in this systematic review and two of them (Clifford et al. [37], Simpson et al. [54]) underwent quantitative analysis for the examined interventions, Capsaicin and Pregabalin respectively. The study of Clifford et al. [37] followed a similar methodology with the older study of Simpson 2008. Pooling of data from these two studies confirmed the efficacious action of Capsaicin 8% in a larger pool of patients. On the other hand, the study of Simpson et al. [54] also followed a similar study design with the older study of Simpson et al. [53]. Authors applied methodological refinements, leading to a study with low risk of bias. Despite these refinements, the non-efficacious effect of Pregabalin also demonstrated in this study and in the pooled analysis of both studies. The study of Dinat et al. [42] examined the effect of Amitriptyline in a cross-over design study. Authors tried to overcome Shlay et al. [32] design drawbacks (where 2x2 factorial design with acupuncture and amitriptyline interventions complicated interpretation). However, Dinat et al. [42] also failed to reveal any positive effect over placebo. Authors raised questions about the median dose of amitriptyline achieved in their study (50 mg per day) as meta-analyses of studies employing amitriptyline for the treatment of neuropathic pain report that the average dose of amitriptyline being taken in trials where amitriptyline was deemed to be superior to placebo was 90 mg per day [57]. Based on the pre-determined criteria for risk of biases, the study was judged as high risk of bias, based on a per protocol analysis (Attrition bias). However, the number of missing participants was too low (one missing subject per group) and probably didn’t account for results inaccuracy.

The study of Harrison et al. [16] evaluated two drugs with quite different mechanisms of analgesic action—duloxetine, and methadone—in a four-period crossover multi-center study. Authors failed to reveal any efficacious effect of either duloxetine, methadone, or their combination, their study suffers from very small sample size, due to premature termination. Based on the fact that both examined pharmacologic agents are for the first time examined in a randomized controlled trial in HIV-SN patients, it is wiser to consider that there are no conclusive data about their effectiveness and safety, rather than definitely preclude any positive action.

In conclusion, although many pharmacologic and non-pharmacologic interventions have been tested for HIV-SN pain, evidence for a positive effect remains rather limited. Most of the RCT are of low quality, are underpowered, and use outcome measurements tool, factors that make it difficult to demonstrate superiority of an intervention with clinical significance. Among different non-pharmacologic interventions, the combination of Acupuncture with Moxibustion (pairing of Acupuncture with moxibustion, the burning of mug wort leaf (Artemisia vulgaris), to stimulate acupuncture points), demonstrated marginally significant pain reduction over placebo, which was maintained after 9 weeks without further treatment in one, high quality RCT. On the other hand, among pharmacologic interventions and after 9 years from Philips et al. [15] meta-analysis, evidence for the positive effect of capsaicin % is supported by a newer study (Clifford et al. [37]). Furthermore, the positive effect of smoked cannabis, already reported by Philips et al. [15] meta-analysis should be underlined in the era of medicinal cannabis legalisation. Other studies (Dinat et al. [42], Harrison et al. [16]) failed to demonstrate any efficacious action for either amitriptyline, methadone, or duloxetine, although conclusive results are difficult to be obtained due to methodological pitfalls of these trials. Finally, the results should be interpreted with caution due to small number of included studies. Indeed, quantitative analysis, whenever was possible to be performed, included up to two studies. During previous years, concerns have been raised regarding the inter-study variance when random-effect model is applied. Inference in random-effects models requires a substantial number of studies included in meta-analysis to guarantee reliable conclusions [58].

## Figures and Tables

**Table 1:** Included studies.

**Adverse events**	No data	No data	16/23 of the LES group: discomfort
**Data**	NRS 0–10 at 12 weeks Median (IQR): AE = 1.0 (1.0), PRE = 1.0 (1.0), control = 3.0 (1.0). AE vs. control, *p* < 0.001, effect size, r = 0.75. PRE vs. control *p* < 0.001, effect size r = 0.83	QOL state of Health: F ratio = 4.24 SE = 0.05, CI:00–0.12 (*p* = 0.04),	Pain scale 0–100: Splint: −32.89 (23.23%), Sham: −14.52% (39.74%), *p* = 935 Pain reduction > 30% Splint 11/18 Liner 5/19
**Outcome measures**	Per protocol analysis Pain intensity and distress NRS 0–10	HR-QOL: ED-5D mobility, EQ-5D self-care, EQ-5D usual activity, EQ-5D pain or Discomfort, EQ-5D anxiety/depression, EQ-5D state of health level	Neuropathic Pain Scale 0–100 The Pittsburgh Sleep Quality Index (PSQI) 0–21
**Intervention**	Aerobic exercise (AE) vs. Progressive resisted exercises (PRE) vs. control. Sessions of 30 min, 3 times/week for 12 weeks	Progressive resisted exercises (PRE) vs. control. Sessions of 30 min, 2 times/week for 12 wee	Walkabout splints or sham (liners only) during night sleep
**ARV therapy**	>6 six months	cART control/PRE: 6–12 months 32/26, 13–24 months 11/9, >25 months 37/45	Splints:20/23 Sham: 20/22
**Sex** **F/M**	AE = 27/18, PRE = 23/21, Control = 27/24	PRE: 57/23 Control: 56/24	Splints: 9/14 Sham: 10/12
**Age**	AE:38.29 (8.06) PRE:35.98 (8.53) Control: 36.13 (8.10)	42.2 (8.5)	Splints: 50.65 (8.04) Sham: 46.09 (8.13)
**Diagnostic criteria**	Referred as diagnosed with HIVDSN	Referred as diagnosed with HIVDSN	Referred as diagnosed with HIVDSN
**Duration**	12 weeks	12 weeks	6 weeks
**Design**	RCT parallel design	RCT parallel design	RCT parallel design
**Participants randomized (completed)**	154 (136)	160 (64)	46 (35)
**Reference**	Maharaj et al. 2018 [30]	Mkandla et al. 2016 [29]	Sandoval et al. 2016 [7]

Abbreviations: HIVDSN = HIV distal sensory neuropathy, AE = Aerobic exercise, PRE = Progressive resiste exercise, QOL = Quality of life; randomized controlled trials (RCTs).

**Table 2:** Included studies.

**Adverse events**	Mild bruising	No data	No data
**Data**	Acu/moxa: 0.85 (SE = 0.12) Sham/control 1.10 (SE = 0.09) *p* = 0.05	BPI average pain intensity Cognitive: −2.6 (3.2) Supportive psychotherapy: −1.3(2.1) (*p >* 0.05)	Vibration therapy: −67.3% (33.4%) Sham therapy: −55% (32%), *p* = 0.92
**Outcome measures**	Daily symptom diary (SD) that incorporated the GPS (0–1.77) Subjective Peripheral Neuropathy Screen (SPNS)	BPI average pain intensity The Brief Symptom Inventory Self-report Beck Depression Inventory Hamilton Depression Rating Scale The Karnofsky Performance Scale	Current-pain item in the Brief Pain Inventory 0 = 10
**Intervention**	Acu/Moxa vs Sham/control	Cognitive behavioural intervention or supportive psychotherapy once weekly	Vibration therapy for 45 min vs. sham therapy for 45 min
**ARV therapy**	Stable regimen for >8 weeks	No data	No data
**Sex** **F/M**	Acu/Moxa: 5/20 Sham/Con: 6/19	13/48	12/28
**Age**	Acu/Moxa: 47.8 (7.2) Sham/control: 47.6 (7.5)	46.5 (7.9)	41.0 (6.0)
**Diagnostic criteria**	Referred as diagnosed with HIVDSN	Referred as diagnosed with HIVDSN	Symmetrical numbness, paraesthesia, or burning, pain ‘now’ score of 4 or greater on BPI 0–10
**Duration**	15 weeks	6 weeks	45 min
**Design**	RCT Parallel design	RCT Parallel design	RCT Parallel design
**Participants randomized (completed)**	50 (50)	61 (33)	40 (40)
**Reference**	Anastasi 2013 et al. [32]	Evans et al. 2003 [31]	Paice et al. 2000a [28]

Abbreviations: HIVDSN = HIV-distal sensory neuropathy, BPI = Brief Pain Inventory.

**Table 3:** Characteristics of included studies.

**Adverse events**	35% of patients stopped drug treatment
**Data**	GPS mean change (14 weeks): SAR (n = 105 −0.29. control (n = 82), −0.19, mean difference −0.08 (CI: −0.21 to 0.06), *p* = 0.26 Amitriptyline (n = 49) −0.26, placebo (n = 52) −30, mean difference 0.00 (CI −0.18–0.19) *p* = 0.99
**Outcome measures**	Primary: Change in GPS between baseline and end of 14 weeks. Secondary: A neurologic summary score, 39-item, qual-ity-of-life assessment tool.
**Intervention**	SAR or control points twice weekly during a 6-week induction phase, followed by weekly treatment during an 8-week maintenance phase. For the amitriptyline comparison, the patients,14-week course of either amitriptyline or placebo capsules 25–75 mg daily
**ARV therapy**	Antiretroviral therapy was allowed
**Sex** **F/M**	SAR:15/106 Control: 10/108 Amitriptyline 5/66 Placebo 8/57
**Age**	Mean (SD): SAR 40.9 (6.8) Control points 41.7 (8.3) Amitriptyline 40.1 (7.1) Placebo 39.9 (5.9)
**Diagnostic criteria**	HIV-related lower extremity peripheral neuropathy, diagnosed by a physician based on history and clinical examination
**Duration**	14 weeks
**Design**	2 × 2 factorial design plus 2 groups active-placebo parallel design
**Participants randomized (completed)**	Factorial Option N = 125 Acupuncture Option N = 114 Amitriptyline Option N = 11
**Reference**	Shlay et al. 1998 [33]

Abbreviations: SAR = standard Acupuncture regimen, GPS = Gracely Pain Scale.

**Table 4:** Pregabalin treatment and treatment-related serious AEs occurred.

**Adverse events**	Discontinuation due to AEs: Pregabalin dizziness (4 subjects), somnolence (2 subjects), confusion state (2 subjects), disorientation (2 subjects). Placebo hypoesthesia (1 subject), bladder pain (1 subject), nausea and vomiting (1 subject), pain (1 subject)	Total AE: Pregabalin = 323, Placebo = 255 Serious AE: Pregabalin = 7, Placebo = 7 Severe AE: Pregabalin = 11, Placebo = 8 Incidence of any AE: Dizziness, Pregabalin = 25 Placebo = 10, Headache Pregabalin = 25, Placebo = 26, Somnolence, Pregabalin = 13, Placebo = 4
**Data**	Pregabalin: −2.88, Placebo: −2.63 (difference −0.25, *p* = 0.3914) 50% responder rate: Pregabalin 38.9%, Placebo 42.8% (*p* = 0.50) 30% responder rate: Pregabalin 56.3%, Placebo 55.9% (*p* = 0.90)	Primary: Change from baseline in LS mean (SE) NRS pain score, Pregabalin: −2.04 (0.15), Placebo: −2.11 (0.15), MD = 0.07 [95% CI = −0.30 to 0.45], *p* = 0.709. Number of patients with >30% response: Pregabalin: 88/183 (48.1%), Placebo: 98/192 (51.0%) OR = 0.84, [95% CI = 0.52 to 1.36], *p* = 0.490
**Outcome** **measures**	Primary: Pain reduction, NRS 0−10. Number of patients with >30% and 50% pain reduction. Secondary: Anxiety, Depression, PGIC	Primary: Pain reduction, NRS 0−10. Number of patients with >30% pain reduction Secondary: PGIC/CGIC, BPI-sf, NPSI. NRS-Sleep scale, MOS Daytime activity and sleep parameters. WPAI-SHP, and SF-36, Hospital Anxiety and Depression Scale HADS
**Intervention**	Pregabalin started at 150 mg/daily, titrated up to 600 mg daily at 2 weeks, stable for next 12 weeks	Pregabalin starting at 150/day, titrated up to 600 mg/day (tolerance and efficacy), during 4 weeks titration period, then maintenance doses for 12 weeks follow up period
**ARV therapy**	Stable doses for >3 months before entry	Stable ARV treatment >8 weeks before the study
**Sex** **F/M**	57/245	Pregabalin: 121/62 Placebo 116/76
**Age**	Mean (SD) Placebo: 46.8 (7.5) Pregabalin: 48.2 (8.1)	Mean (SD) Pregabalin: 41.2 (9.0) Placebo: 42.3 (8.4)
**Diagnostic criteria**	clinical	2 of the 3 following signs: reduced or absent Achilles tendon reflexes, superficial and vibratory sensation in the lower extremities, daily pain (>40 mm on the VAS [range 0–100] scale
**Duration**	14 weeks	16 weeks
**Design**	Randomized parallel group	Randomized parallel group
**Participants randomized (completed)**	302 (299)	377 (375)
**Reference**	Simpson et al. 2010 [34]	Simpson et al. 2014 [35]

Abbreviations: ARV = Antiretroviral therapy, (PGIC/CGIC) = Patient and Clinician Global Impression of Change, BPI sf = Brief Pain Inventory short form, NPSI = Neuropathic Pain Symptom Inventory, MOS = Medical Outcomes Survey.

**Table 5:** Cannabis and treatment.

**Adverse events**	Cannabis: Severe dizziness: 1 episode Anxiety: 1 episode Placebo: Anxiety: 1 episode. Confusion, dizziness, nausea significantly more frequent in cannabis group (*p* < 0.01). No withdrawals due to adverse events	Greater frequency of concentration difficulties, fatigue, sleepiness or sedation, increased duration of sleep, reduced salivation, and thirst in cannabis week than placebo week
**Data**	Primary outcome 1. >30% pain change Cannabis, 13/25 patients, placebo: 6/25 patients (52% vs. 24%, difference 28%, 95% CI 2%–54%, 2. Median change in pain (VAS): Cannabis −34% (IQR −71, −16) placebo −17% (IQR −29, +8) dif 18%, *p* = 0.03. Secondary: Painful area brush and von Frey hair stimuli: Cannabis median −34%, −52% vs. Placebo −11%, +3% respectively *p* = 0.05	Primary: Median difference in pain reduction = 3.3 favouring cannabis, Effect size = 0.60, *p* = 0.020 (ITT). First week: DDS Median change: cannabis −4.1, placebo +0.1 *p* = 0.029. Proportion of patients with >30 DDS pain reduction: cannabis week 0.46 (95%CI 0.28,0.65), placebo week 0.18 (0.03, 0.32), *p* = 0.043 VAS median change (range) Cannabis −17 (−58, 52) placebo −4 (−56, 28), (*p* < 0.001)
**Outcome** **measures**	Primary: 1. Proportion of patients with >30% reduction in pain from baseline to end of treatment 2. The percent change in pain from baseline (VAS) Secondary: 1. Percent change after 1st and last cigarette in pain, and secondary hyperalgesia 2. Change in total mood disturbance (Profile of Mood States)	Primary: 1. Change in self-reported pain magnitude assessed by the DDS (0- to 20-point scale). 2. Change in VAS scale (0–10) Secondary: Disability, mood, and quality of life (Sickness Impact Profile (SIP), Profile of Mood States (POMS) the Brief Symptom Inventory (BSI)
**Intervention**	Cannabis cigarettes smoking (3.56% delta-9-THC) or placebo cannabis cigarettes (0% delta-9-THC), 3 times daily for 5 consecutive days	4 smoking sessions/day for 5 consecutive days with active (Δ-9 ΤHC 1–8%, titrated to effect) or matching placebo cigarettes, 2 weeks washout followed by another 4 smoking sessions/day for another 5 days (placebo or active)
**ARV therapy**	Stable regimen for at least 8 weeks prior to randomization: Cannabis: 18/27 Placebo: 26/28	Prescribed: 93% Exposed to potentially neurotoxic dideoxy- nucleoside reverse transcriptase inhibitors: 72%
**Sex** **F/M**	Cannabis 5/22 Placebo 2/26	0/34
**Age**	Mean (SD) Cannabis 50 (6) Placebo: 47 (7)	48.8 (6.8)
**Diagnostic criteria**	Symmetric distal pain or dysesthesias > 2 weeks, absent or depressed ankle reflexes, or pin, vibration, touch, temperature sensory loss	Reduced distal tendon reflexes, distal sensory loss or electro-physiological abnormalities (distal leg sensory nerve conduction studies), plus symptoms of pain and paraesthesias
**Duration**	5 days	5 days, 2 weeks washout cross over to another 5 days
**Design**	Randomized parallel group	Single group, double-blind, placebo-controlled crossover
**Participants randomized (completed)**	55 (50)	34 (28)
**Reference**	Abrams et al. 2007 [36]	Ellis et al. 2009 [37]

Abbreviations: THC = tetrahydrocannabinol, VAS = visual analog scale.

**Table 6:** Primary effective outcome.

**Adverse events**	NGX-4010: 161/225 control: 45/82 Dropouts due to adverse events: NGX-4010, n = 2; control, n = 1.	% of patients with >1 AE: 93% NGX-4010, 83% control group.	Drop out: Capsaicin cream n = 5 (burning)
**Data**	NRPS scores from baseline to week 12, Mean (SD): NGX 4080 −22.8% (30.6), control −10.7% (30.8), *p* = 0.0026 >30% change in pain: NGX 4080 76/225, control 15/82	Change from baseline to weeks 2–12, mean (SE): NGX 4010 −1.8 (0.1) Control, −1.4 (0.2), *p >* 0.05. >30% pain reduction: NGX-4010 43/332, control 36/162 *p >* 0.05	BPI at study endpoint Mean (SD); capsaicin 5.50 (2.68) Control 3.10 (2.12), *p* = 0.042
**Outcome** **measures**	Primary: % change in the pain NPRS score, from baseline to weeks 2 to 12. Secondary: Change from baseline pain for weeks 2–4 and 2–8; proportion of patients with a >30% mean decrease “average pain; percent change from baseline in the “worst pain for the past 24 h” and “pain now”. Change from baseline to week 12 assessed with GPS sf McGill, BPI, PGIC and CGIC	Primary: percent change in NPRS scores from baseline during weeks 2–12, patients with a >30% average pain reduction, the percentage of patients improved on PGIC and CGIC, changes from screening in sfMcGill Pain Questionnaire and SF-36v2	Change in BPI, Quality of Life Index (QLI), Profile of Mood States (POMS), Sickness Impact Profile (SIP)
**Intervention**	NGX-4010 (capsaicin 640 mcg/cm^2^, 8% *w*/*w*) Patch or control patch (patch (3.2 mcg/cm^2^, 0.04% *w*/*w*) applied for 30, 60, or 90 min, up to 4 patches each	NGX-4010 (capsaicin 640 mg/cm^2^, 8% *w*/*w*; or control capsaicin (3.2 mg/cm^2^, 0.04% *w*/*w*) patch, for 30 or 60 min to both feet (up to 1120 cm^2^)	Topical capsaicin (0.075%) plus usual therapy, or placebo plus usual therapy, 4 times daily for 4 weeks
**ARV therapy**	No ART or on stable doses for >8 weeks	Exposure NGX-4010: 25/332, control: 8/162	No use of didenine or didectosine
**Sex** **F/M**	NGX 4010: 18/207 Control 3/79	NGX 4010: 42/332 Control 20/162	1/25
**Age**	Mean (SD): NGX-4010 47.7 (8.4) Control 48.4 (7.6)	Mean (SD) NGX-4010 49.7 (8.5) Control 49.7 (8.7)	Mean (SD): 40.3 (6.0)
**Diagnostic criteria**	Pain, burning, dysesthetic discomfort in both feet, diminished ankle reflexes, and diminution of vibration, pain, or temperature sensation in the distal legs	Diagnosed with HIV-DSP for >2 months and an average baseline numeric pain rating scale (NPRS) score of 3–9	Diagnosed HIV-related DSPN
**Duration**	12 weeks	12 weeks	4 weeks
**Design**	Randomized-controlled parallel group	Randomized-controlled parallel group	Randomized-controlled parallel group
**Participants randomized (completed)**	307 (274 completed, 302 analysed)	494(234)	26 (14)
**Reference**	Simpson et al., 2008 [39]	Clifford et al., 2012 [38]	Paice 2000b

Abbreviations: DS*P* = distal sensory polyneuropathy, ART = antiretroviral therapy, NPRS = Numerical Pain rating scale.

**Table 7:** Lamotrigine outcome.

**Adverse events**	Drop out: Lamotrigine, rash n = 5, gastrointestinal infection n = 1	Rash: Lamotrigine 21/150 Control 9/77 Infection 17/150, control 7/77, Nausea: Lamotrigine 17/150, control 8/77 Diarrhea Lamotrigine: 16/150, control 7/77 Headache: Lamotrigine 16/150, control 8/77
**Data**	Mean difference (SE) pain scores between baseline and week 14: Lamotrigine −0.55 (0.14), control −0.18 (0.09), *p* = 0.03. No difference in global pain score (*p* = 0.37), worst pain score (*p* = 0.17), or change in use of concomitant analgesics (*p* = 0.99) at week 14 between the two groups	Neurotoxic striatum, mean change: Lamotrigine: −0.03, control −0.007, *p* < 0.05 Non neurotoxic striatum, mean change: Lamotrigine −0.022, control −0.0025, *p >* 0.05
**Outcome** **measures**	Primary: Pain reduction measured by GPS. Safety and Tolerability Secondary: The slope of change of weekly mean pain scores over course of trial. Patient-rated global pain relief, change in worst pain, use of concomitant analgesic medications	Primary efficacy measure: the mean change in average pain (GPS up to the maintenance phase (PP analysis) Secondary efficacy endpoints: analysis of slope of weekly average GPS, the mean change in pain (VAS, McGill Pain Questionnaire), the percentage of patients with >30% VAS change
**Intervention**	Lamotrigine or patching placebo titrated up to 150 mg × 2 up to 7 weeks, then stable for 7 more weeks	Lamotrigine or matching placebo titrated up to 600 mg daily for 7 weeks, then stable dose for 4 weeks
**ARV therapy**	No neurotoxic antiretroviral therapy for >8 weeks before randomization or a history of a stable dose for at least 8 weeks before randomization	No prior exposure to ddX ART, stop them >8 weeks or at stable dose for >8 weeks before randomization.
**Sex** **F/M**	Lamotrigine: 1/8. Control 4/16 (completers)	Lamotrigine 15/150 Control 11/77
**Age**	Lamotrigine 44.6 (8.4) Control 44.4 (10.6) (completers)	Mean (range) Neurotoxic stratum: Lamotrigine 44 (32–65), placebo 42 (29–67) No neurotoxic striatum: Lamotrigine45 (26–63), placebo 46 (33–64)
**Diagnostic criteria**	Symptoms of burning or dysesthetic pain in both feet for at least 2 weeks, rated on the GPS as at least “mild” all of the time or moderate” for a total of at least 2 hours a day, and either absent or diminished ankle reflexes or distal diminution of either vibration sensation or pain and temperature sensation	Symptoms of neuropathic pain in both distal lower extremities plus either diminished reflexes at the ankles distal diminution of sensations of vibration, pain, or temperature in the legs.
**Duration**	14 weeks	12 weeks
**Design**	Randomized parallel group	Randomized parallel group
**Participants randomized (completed)**	42 (28)	227 (172)
**Reference**	[40] and [41]	Simpson et al. 2003

Abbreviations: GPS = Gracely Pain Scale, ddX: dideoxynucleoside analogue.

**Table 8:** Amitryptiline and Mexiletine outcome.

**Adverse events**	Dry mouth: Amitript = 9, placebo n = 1; Drowsiness: Amitriptyline n = 7, placebo n = 1; Chest pain: Placebo n = 1	Amitriptyline (sedation n = 10, confusion n = 1, less common events n = 4), Mexiletine (nausea and vomiting n = 10, urinary retention n = 3, dizziness n = 1, other n = 8) Placebo (confusion n = 21, urinary retention n = 1, other n = 3)	Drug stop: Mexiletine, 1 rush, 2 gastrointestinal side effects. 1, ECG changes
**Data**	Primary: ARV users: amitriptyline: −2.7, SD −3.3; placebo: −2.1, SD −2.8; t(60) = −1.13, *p* = 0.26 ARV naïve: amitriptyline: −2.8, SD −3.3; placebo: −2.8, SD −3.4; t(60) = 0.05, *p* = 0.96	Amitriptyline group (n = 39): Mean −0.31 (SD 0.31). Mexiletine group (n = 44): Mean −0.23 (SD 0.41). Placebo group (n = 43): −0.20 (SD 0.30), *p* = 0.38. The mean reduction in pain intensity with Amitriptyline, relative to placebo: −0.11	Mean pain scores (SD): First Mexiletine 30.8 (16.1), then placebo: 34.0 (29.6) *p* = 0.78. First placebo 54.2 (19.5) then Mexiletine 45.7 (27.3), *p* = 0.45
**Outcome** **measures**	Per protocol analysis Primary: Likert [0–10]: Difference in pain intensity between baseline and at six weeks. Secondary: Dose escalation and maximum dosage of amitriptyline Side effects and adverse events. The use of rescue medication	Primary: GPS [0–1.77]: Change in mean pain intensity from baseline to week 8. Safety: clinical adverse events, and laboratory test abnormalities, dosage modification caused by adverse events Secondary: Changes in mood, quality of life, requirement for additional analgesic agents	Primary: Pain reduction, VAS 0–100 Secondary: adverse events
**Intervention**	Amitriptyline vs. placebo (6 weeks, median dose = 50 mg)	Amitriptyline + placebo Mexiletine, placebo Amitriptyline + Mexiletine, placebo Amitriptyline + placebo Mexiletine. 4 weeks titration, 4 weeks stable dose, up to 600 mg Mexiletine and 100 mg Amitriptyline	Mexiletine up to 600 mg/day vs. placebo for 6 weeks, 1 week washout then Placebo vs. Mexiletine
**ARV therapy**	Stable therapy for>6 months (ARV user group), or therapy naïve (ARV-naïve group). ARV-naïve (n = 61) ARV-user (n = 61)	Current use: n = 49, Discontinued 8-26 weeks prior to study: n = 35, Never used/discontinued > 26 weeks prior to study: n = 61	No early use of ddI, ddC within one year
**Sex** **F/M**	87/35	6/139	2/20
**Age**	Mean (SD) 38 (8.9)	Median: Amitriptyline39 Mexiletine 40	Mean:35
**Diagnostic criteria**	Brief Peripheral Neuropathy Screening Tool	Symmetrical pain, burning or tingling at least mild all the time or moderate for >2 h/day and diminished ankle reflexes or distal diminution of vibratory sense or diminished pain and temperature sensation	Pain >4/10 in VAS, decrease in pinprick or vibratory sense, decrease or absent ankle jerks
**Duration**	15 weeks	8 weeks	6 weeks, one week washout then another 6 weeks
**Design**	Randomized cross-over	Randomized parallel	Randomized cross-over
**Participants randomized (completed)**	124 (122)	145 (126)	22 (19)
**Reference**	Dinat et al., 2015 [43]	Kieburtz et al., 1998 [44]	Kemper et al., 1998 [45]

Abbreviations: VAS = Visual Pain Scale, ddI = didanosine, ddC = zalcitabine.

**Table 9:** Gabapentin and Recombinant human nerve growth factor (NGF).

**Adverse events**	GBP-group: 80% patients dizziness and significantly more frequent than placebo patients *p* < 0.05. Dizziness, gait ataxia and nausea were more frequent in the GBP-group, but not statistically significant compared with placebo patients	Side effects: 22 patients low dose NGF. 56 patients NGF, 25 patients placebo group. Most frequent injection site pain or myalgias on all groups
**Data**	GBP: median baseline week VAS = 5.1, median 4th week VAS = 2.85, −44.1%, *p* < 0.05 Placebo: median baseline week VAS = 4.7, median 4th week VAS = 3.3, −29.8%, *p* = 0.646). Comparison of the changes between GBP and placebo-group: no significant differences for the pain or the sleep interference score.	The mean adjusted change: Placebo, −0.06 [range −0.14 to +0.01 log units] 0.1 μg/kg rhNGF: 0.18 (−0.25 to −0.1 log units] 0.3 μg/kg rhNGF: 0.21 [−0.29 to −0.14log units]
**Outcome** **measures**	Primary outcome: Pain change (10-cm VAS of SF-MPQ). Primary endpoint: Difference in weekly median pain score between the 4th week and the baseline week. Secondary: median sleep interference score, measured by VAS (0 = excellent sleep, 10 cm = no sleep)	Primary endpoint: Change in pain intensity (GPS) from baseline to week 18. Secondary: Global assessments of neuropathic pain
**Intervention**	Gabapentin dosage and matching placebo titrated over 2 weeks up to 1200 mg/d. In the case of sufficient, effect the dosage was increased up to 2400 mg/d over further 2 weeks	0.1 mg/kg rhNGF s.c. 2 times/week, 0.3 mg/kg rhNGF s.c. 2 times/week, placebo s.c. 2 times/week
**ARV therapy**	7 patients with concomitant antiretroviral treatment of d4T and/or ddI (GBP n = 4; placebo n = 3) and 3 patients, who had had neurotoxic antiretroviral drugs (d4T and/or ddI) in the period of 3 months before he study (GBP n = 2; placebo n = 1)	Subjects stratified regarding ddI, ddC, or d4T use: current use, use stopped 8–26 weeks before randomization stopped >26 weeks before randomization, never used
**Sex** **F/M**	6/20	8/262
**Age**	Median (range) GBP: 46 (27–59) Placebo: 44 (35–61)	Mean (SD): 44.0 (8.7)
**Diagnostic criteria**	Based on history, clinical and neurophysiological examination (paraesthesia, dysesthesia or pain), abnormal sensory signs (elevated vibratory threshold or pin hyperalgesia), decreased or absent ankle reflexes.	Clinical, based on criteria set by the American Academy of Neurology
**Duration**	4 weeks	18 weeks
**Design**	Randomized, double blind, parallel group	Randomized, double blind, parallel group
**Participants randomized (completed)**	26 (24)	270 (235)
**Reference**	Hahn et al. 2004 [46]	McArthur et al., 2002 [47]

Abbreviations: GB*P* = Gabapentine, d4T = stavudine, ddI = didanosine, ddC = zalcitabine, rhNGF = recombinant Nerve Growth Factor, GPS = Gracely Pain Scale, GPB = Gabapentin, SF-MPQ = Short Form McGill Pain Questionnaire.

**Table 10:** Acetyl L-carnitine and lidocaine.

**Adverse events**	23 patients with 1 or more AE; ALCAR n = 1 (20.9%) Placebo: n = 14 (29.8%) Events related to study medication ALCAR group: paraesthesia, 1 subject; pain, anorexia, dry mouth and neuropathy, 1 patient. Placebo group:4 patients diarrhoea, nausea, pruritus and rash)	Lidocaine gel: local rash, blisters, and dryness, n = 3
**Data**	Primary: VAS reduction Mean (SD) ALCAR–1.32 (1.84) Placebo–0.61 (1.55), *p* = 0.07 2. Secondary: TSS change Mean (SD): ALCAR -1.32 (2.45) Placebo −0.88 (1.90), *p* = 0.19 Proportion of patients with >30% improvement in TSS: 30.2% Placebo: 18.2%, *p* = 0.21	Primary outome: Pain scores at end of phase A: Mean (SD) Lidocaine 1.09 (0.24) Placebo 1.15 (0.32), difference at end of Phase A: Lidocaine 0.03 (0.23), placebo 0.08 (0.16), *p* = 0.314 Pain scores at end of phase B: Mean (SD) Lidocaine 1.16 (0.33) Placebo 1.10 (0.32), difference at end of Phase B: Lidocaine 0.11 (0.23), placebo 0.00 (0.13), *p* = 0.744
**Outcome** **measures**	Primary: Pain change (VAS) between baseline and 14 days. Secondary: Total Symptom Score (TSS), (CGI-C), MPQ, need for rescue analgesics	Primary outcome: Difference in GPS pain scores during the 2nd week of each period. Secondary analyses:(1) differential response of first treatment, (2) global pain relief (3) effect of exposure to nucleoside analogue on the response to lidocaine gel
**Intervention**	ALCAR 1000 mg/day or placebo i.m., 2 times/day for 14 days	Active gel (5% lidocaine gel) or vehicle placebo gel, applied once daily for 2 weeks. They were then crossed over to the second 2-week treatment period on the alternate drug
**ARV therapy**	Stable ATN (onset within 6–12 months of commencing NRTI therapy, symptoms stable for 42 months, and no other neuropathic aetiological factors or DSP-associated therapies)	Current stable use: 21 not used for the previous 8 weeks: 41
**Sex** **F/M**	18/72	No data
**Age**	44.4 (9.8)	45
**Diagnostic criteria**	Electrophysiological diagnosis	Presence of pain or paraesthesias in both feet for at least 2 weeks, rated on the GPS as at least mild all the time or moderate for a > 2 h/day and diminished or absent ankle reflexes, or pain, temperature, or vibration sensation in the legs
**Duration**	14 days	6 weeks: 2 weeks Phase A, 2 weeks washout, 2 weeks Phase B
**Design**	Randomized controlled parallel group	Randomized controlled cross-over study
**Participants randomized (completed)**	90 (87)	64 (56)
**Reference**	Youle et al. 2007 [48]	Estanislao et al. 2004 [49]

Abbreviations: ATN = antiretroviral toxic neuropathy, NRTI = Nucleoside reverse transcriptase inhibitors, Acetyl-L-carnitine, TTS = Total Symptom Score, CGI-C = Clinical Global Impression of Change, MPQ = McGill Pain Questionnaire.

**Table 11:** Peptide T and Prosaptide.

**Adverse events**	No differences between groups. One patient in placebo group: mild epistaxis	PRO: 4 AE (cellulitis, altered mental status, higella enteritis, pancreatitis) PBO: 1 AE (Kaposi sarcoma)
**Data**	Primary (PP analysis): Pain score differences between baseline and week 12, Mean (SD): Peptide T: −0.24 (0.45) Placebo: −0.39 (0.54), *p* = 0.3	GPS changes mean (SD): PRO 2 mg/day −0.12(0.23) PRO 4 mg/day −0.24(0.35) PRO 8 mg/day −0.15(0.32) PRO 16mg/day −0.18(0.34) PBO −0.18(0.32), p.0.05 between all comparisons
**Outcome** **measures**	Primary: Reduction in pain severity (GPS) at week 12 Secondary: were neurologic examination, nerve conduction studies, global evaluation, electrophysiologic measurements, cognitive function and immunological function	Primary efficacy endpoint: change from baseline to 6 week endpoint GPS weekly average. Secondary endpoints: defined as >0.35 units of pain improvement from baseline on the GPS, change in HIV viral load
**Intervention**	Peptide T 6mg intranasally/ day or placebo intranasally for 12 weeks	2, 4, 8, or 16 mg/d PRO or PBO administered via S.C. injection for 6 weeks
**ARV therapy**	No use of use of zidovudine (ZDV, AZT) for less than three months before entry, no use of didanosine (ddI) and/or zalcitabine (ddC) within eight weeks of entry Current use of Zidovudine: Peptide T, n = 28 Placebo, n = 35	Stable use or non-use of dideoxynucleoside reverse transcriptase inhibitors for >4 months. ddC, d4T, or ddI use at Entry: 52/229
**Sex** **F/M**	Peptide T:38/2 Placebo 39/2	19/210
**Age**	Median: Peptide T = 40.4 Placebo = 40.9	Median, Q1, Q3: 47,43,53
**Diagnostic criteria**	(1) Distal pain, paraesthesia, or numbness, of the lower extremities. (2) Neurologic signs, (reduction in pain, temperature, touch, or vibratory sensation in a stocking and glove distribution; absent or reduced ankle reflexes. (3) Electrophysiologic signs of generalized, distal, sensory and motor, axonal polyneuropathy	Clinical criteria developed by the American Academy of Neurology (1991)
**Duration**	12 weeks	6 weeks
**Design**	Randomized, placebo-controlled, parallel design study	Randomized, placebo-controlled, 4 arm parallel design study
**Participants randomized (completed)**	81 (75)	237 (196)
**Reference**	Simpson et al. 1996 [50]	Evans et al. 2007 [51]

Abbreviations: ZDV, AZT = Zidovudine, ddI = didanosine, ddC = zalcitabine, PRO = Prosaptide, P*P* = Per protocol, PBO = Placebo, GPS = Gracely pain scale.

**Table 12:** Memantine, Duloxetine, Methadone.

**Adverse events**	No differences between groups	Adverse events: Duloxetine, n = 5 Methadone, n = 17 Conbination, n = 17 Placebo, n = 6 Severe adverse events: Duloxetine nausea (n = 1), vomiting (n = 1), renal dysfunction (n = 1). Severe adverse events on placebo: pain (n = 1), fatigue (n = 1).
**Data**	Primary, Pain change Mean (SD) Memantine = −1.82 (2.77) Placebo = −2.36 (3.35), *p* = 0.87 Change of the paresthesia score, mean (SD): Memantine = −0.91 (3.58) Placebo = −1.14 (3.35), *p* = 0.92	Primary: 4th week pain scores (median Q1, Q3) A. Dul/Placebo:7 (4, 8) B. Placebo/Placebo: 6 (4, 8) C. Placebo/Meth: 6.5 (5, 8) D. Dul/Meth: 5.5 (4, 7). Comparisons A vs. B, *p* = 1, C vs. B, *p* = 1, D vs. B, *p* = 0.25, D vs. A, *p* = 0.11, D vs. C, *p* = 0.06
**Outcome** **measures**	Primary: change in pain and paraesthesia indices on a 01–10 scale, from baseline to week 16, between memantine and placebo arms.	Primary outcome measure: mean 24 h pain intensity (MPI) measured on 0–10 NRS. Secondary Outcome: night-time pain intensity
**Intervention**	Memantine starting at 10 mg/day, titrated up to 40 mg in 4 weeks (or up to the maximum tolerated dose, stable up to week 16.	Patient assigned to one of 4 to one of four treatment sequences, including Duloxetine, Methadone, Duloxetine-Methadone or placebo, 4 weeks each with one week washout. Duloxetine/matching placebo titrated to 60mg. Methadone/matching placebo titrated to 10 mg t.i.d.
**ARV therapy**	Memantine: 2/24 Placebo 20/21	Stable use or non-use of antiretrovirals for 30 days prior to entry
**Sex** **F/M**	Memantine2/22 Placebo: 4/17	2/13
**Age**	Median (min, max): Memantine group: 44 (33, 63) Placebo group: 46 (31, 59)	13/15 over 50 years
**Diagnostic criteria**	Presence of symmetric loss or reduction of vibratory, pinprick, or temperature sensation in a stocking and glove distribution and predominantly symmetric pain or paraesthesia	Presence of symmetrical pain, burning, or dysesthesias in a stocking distribution for at least 6 months with abnormal ankle reflexes or at least one abnormal sensory sign (elevated vibratory thresholds, stocking loss of pinprick or temperature, or cutaneous allodynia)
**Duration**	16 weeks	20 weeks
**Design**	Randomized, double blind, placebo-controlled, parallel study	Randomized, double blind, placebo-controlled, four-period crossover study
**Participants randomized (completed)**	45	15 (8)
**Reference**	Shiffito et al. 2006 [52]	Harrison et al. 2013 [53]

Abbreviations: Dul = Duloxetine, Meth = Metadone.

## Appendix A

Searching strategy for PUBMED

**((((((hiv) OR human immunodeficiency virus) OR aids)) AND (((((pain) OR painful) OR analg*) OR nocicept*) OR antinocicept)) AND (((neuropathy) OR polyneuropathy) OR neuropathic)) AND (random*)**
